# Comparisons of oral, intestinal, and pancreatic bacterial microbiomes in patients with pancreatic cancer and other gastrointestinal diseases

**DOI:** 10.1080/20002297.2021.1887680

**Published:** 2021-02-14

**Authors:** Mei Chung, Naisi Zhao, Richard Meier, Devin C. Koestler, Guojun Wu, Erika del Castillo, Bruce J. Paster, Kevin Charpentier, Jacques Izard, Karl T. Kelsey, Dominique S. Michaud

**Affiliations:** a Department of Public Health and Community Medicine, School of Medicine, Tufts University, Boston, MA, USA; b Department of Biostatistics, University of Kansas Medical Center, Kansas City, KS, USA; c University of Kansas Cancer Center, The University of Kansas Medical Center, Kansas City, KS, USA; d Department of Biochemistry and Microbiology, Center for Nutrition, Microbiome and Health, New Jersey Institute for Food, Nutrition and Health, Rutgers University, New Brunswick, NJ, USA; e Department of Microbiology, The Forsyth Institute, Cambridge, MA, USA; f Department of Oral Medicine, Infection & Immunity, Harvard School of Dental Medicine, Boston, MA, USA; g Department of Surgery, Rhode Island Hospital, Providence, RI, USA; h Department of Food Science and Technology, University of Nebraska, Lincoln, NE, USA; i Fred and Pamela Buffet Cancer Center, University of Nebraska Medical Center, Omaha, NE, USA; j Center for Environmental Health and Technology, Brown University, Providence, RI, USA

**Keywords:** Oral microbiomes, intestinal microbiomes, gastrointestinal diseases, pancreatic cancer

## Abstract

**Background**: Oral microbiota is believed to play important roles in systemic diseases, including cancer. **Methods**: We collected oral samples (tongue, buccal, supragingival, and saliva) and pancreatic tissue or intestinal samples from 52 subjects, and characterized 16S rRNA genes using high-throughput DNA sequencing. **Results**: Bray–Curtis plot showed clear separations between bacterial communities in the oral cavity and those in intestinal and pancreatic tissue samples. PERMANOVA tests indicated that bacterial communities from buccal samples were similar to supragingival and saliva samples, and pancreatic duct samples were similar to pancreatic tumor samples, but all other samples were significantly different from each other. A total of 73 unique Amplicon Sequence Variants (ASVs) were shared between oral and pancreatic or intestinal samples. Only four ASVs showed significant concordance, and two specific bacterial species (Gemella morbillorum and Fusobacterium nucleatum subsp. vincentii) showed consistent presence or absence patterns between oral and intestinal or pancreatic samples, after adjusting for within-subject correlation and disease status. Lastly, microbial co-abundance analyses showed distinct strain-level cluster patterns among microbiome members in buccal, saliva, duodenum, jejunum, and pancreatic tumor samples. **Conclusions**: Our findings indicate that oral, intestinal, and pancreatic bacterial microbiomes overlap but exhibit distinct co-abundance patterns in patients with pancreatic cancer and other gastrointestinal diseases.

## Introduction

The oral cavity is a major gateway to the human body. It is estimated that the oral cavity collectively harbors over 700 predominant bacterial species [1]. Oral microbes have been shown to contribute to a number of oral diseases, including tooth caries, periodontitis, endodontic infection, alveolar osteitis, and tonsillitis. It is hypothesized that oral opportunistic or pathogenic bacteria can enter into the blood circulation, passing through the oral mucosal barrier, potentially resulting in abnormal local and systemic immune and metabolic responses [2]. Oral microbiota have been shown to play important roles in systemic diseases such as cardiovascular diseases, diabetes mellitus, respiratory diseases, and cancer [3–6].

Many studies have investigated the relationship between the oral or gut microbiome and various cancer risks using different methods and study designs [7,8]. Among these, colorectal cancer (CRC) is the most studied cancer. The unexpected finding that species of *Fusobacterium*, particularly the oral species *Fusobacterium nucleatum*, are very prevalent (about 30%) in CRC cases suggested an association between the oral microbiota in the colon and CRC [8]. Research on the relationships between oral bacteria and pancreatic cancer risk stems from a number of observational studies that have reported a higher risk of pancreatic cancer among individuals with periodontitis, when compared to those without periodontitis [9–12]. A number of studies have examined the association of the oral microbiome with pancreatic cancer risk [13–16], but results were inconsistent partially due to differences in methods and study designs. Although one recent study found suggestive evidence that oral dysbiosis is a causative effect of early pancreatic cancer [13], more prospective studies are needed to replicate and confirm their findings. Defining oral microbial profiles as non-invasive biomarkers for pancreatic cancer could help screening of high-risk populations. However, no human study to date has correlated microbiome in the oral cavity with the microbiome in pancreatic tissue of the same patients.

In an effort to address the specific question of whether the pancreas has its own microbiome, we recruited subjects with planned foregut surgery to obtain pancreatic tissue samples for 16S rRNA gene microbiome analysis. The characteristics of the overall bacterial microbiome in pancreatic and normal surrounding tissue samples have been reported elsewhere [17]. Briefly, we reported that bacterial taxa known to inhabit the oral cavity, including several putative periodontal pathogens, were common in the pancreas microbiome. Moreover, bacterial DNA profiles in the pancreas were similar to those in the duodenum tissue of the same subjects, regardless of disease state, suggesting that bacteria may disseminate from the gut into the pancreas [17]. Since oral samples were also collected from these patients, the present study provides a unique opportunity to examine the oral microbiome profiles (at multiple oral cavity sites) and their correlations with the microbiome in pancreatic tissue and intestinal tissue or surfaces. Additionally, we aimed to characterize and compare the microbial communities at different oral sites among these patients with pancreatic cancer and other gastrointestinal diseases. Bacterial co-abundance networks were also plotted to visualize the overall community at different sampling sites.

## Methods

### Sampling, DNA extraction, and 16S rRNA gene amplicon sequencing

Based on sample-availability, in the present study subjects who underwent surgery for pancreatic diseases or diseases of the foregut, at the Rhode Island Hospital between 2014 and 2016 and contributed pancreatic tissue (pancreatic duct, normal or tumor pancreas) or intestinal (duodenum tissue, jejunum swab, bile duct swab) sample was analyzed. Details of the study population and sample collection, DNA extraction, and sequencing procedures have been described in our previous publication [17] and in Supplemental Material 1. Briefly, data on participants’ demographics and behavioral factors were collected using a self-administered questionnaire, and pancreatic tissue samples and gastrointestinal swabs were collected during surgery using DNA-free forensic sterile swabs whenever possible to reduce contamination. Oral swabs were collected from participants prior to surgery using sterile cytology brushes which were immediately placed in tubes containing 700 μl RNA later solution after collection. Saliva was collected using saliva kits (OMNIgene OM-501, DNA Genotek) and processed as per manufacturers’ instructions. All samples were de-identified and stored at −80°C until processing for DNA extraction. Hypervariable regions of the 16S rRNA gene were sequenced using primers targeting the V3-V4 as paired-end reads on an Illumina platform. Subjects contributing only stomach swab, ileum swab, and pancreatic swab samples were excluded from the present analyses due to the small number of samples available.

Sequence quality checking and denoising were performed using the DADA2 Illumina sequence denoising process [18]. Human-associated DNA contaminants were screened out using Bowtie2 [19]. Taxonomic classification, alignment, and phylogenetic tree building were completed using the Quantitative Insights Into Microbial Ecology version 2 (QIIME2) [20,21]. The sequences of each sample were rarefied to 1200 to even the difference in sequencing depth across both oral and intestinal samples for further analysis. The choice of 1200 as sampling depth was guided by reviewing an alpha rarefaction curve that tested various depths ranging between 500 and 5,000 (Figure in the Supplemental Material 1).

### Ethics approval and consent to participate

The study was approved by Lifespan’s Research Protection Office for recruitment at RIH, as well as the Institutional Review Boards for Human Subjects Research at Brown University, Tufts University, and the Forsyth Institute. A written informed consent was obtained from all subjects. All methods carried out were in accordance with Helsinki Declaration as revised in 2013.

### Assigning taxonomic annotation

To predict the taxonomic groups that are present in each sample, the QIIME2 plugin (q2-feature-classifier) was used to train naïve Bayes classifiers using multiple databases as a different set of reference sequences. These were the Human Oral Microbiome Database (HOMD) (version 15.1), the Silva (release 132), and the Greengenes (13_8 revision) 99% OTUs (Operational taxonomic units) 16S rRNA gene databases, all trimmed to contain the V3-V4 hypervariable region. HOMD identification was chosen as the default taxonomy. Whenever HOMD, Silva, and Greengenes yielded different taxonomic results at the species level, taxonomic information from all datasets were kept and reported.

### Statistical analyses

Analyses were carried out in QIIME2 (https://qiime2.org), in R [22], and in Python. All analyses were conducted using ASV as the unit of observation in order to accurately capture bacterial strain-level variations.

#### Alpha and beta diversity

For the calculation of alpha and beta-diversity measures of the oral microbiome, a phylogenetic tree was first created in order to generate phylogenetic diversity measures such as Faith’s Phylogenetic Diversity, unweighted and weighted UniFrac distances [23,24]. Creating a phylogenetic tree requires multiple sequence alignment, masking, tree building, and rooting. The masking step removes alignment positions that do not contain enough conservation to provide meaningful information (default 40%). Next, the evenness and diversity of oral microbiota in each sample was assessed to examine the variation in the microbial profile across different oral sampling sites. Pielou’s Evenness test and Faith’s Phylogenetic Diversity test were calculated using the QIIME2 diversity analyses (q2‐diversity) plugin [25]. Computed distances were then used to generate principal coordinate analysis (PCoA) plots to visualize the arrangement of the samples in the ordination space. PERMANOVA tests [26] were conducted to compare beta-diversity measures between sampling sites. A false discovery rate (FDR) adjusted p-value (or q-value) less than 0.05 was considered the significant difference in beta-diversity measures between oral sites.

#### Identifying shared ASVs

Descriptive analyses were performed to identify shared ASVs between sites. Rarefied features with a relative abundance less than or equal to 0.01 (≤1%) were set to zero. Shared ASVs between any one of the oral sites (tongue, buccal mucosa, supragingival, or saliva) and any pancreatic tissue or intestinal sites (duodenum tissue, jejunum swab, bile duct swab) for each subject were identified. A heatmap of the ASVs that were shared by one or more subjects was generated in R using the packages *intersect* and *ggplot2*.

#### Concordance and Pairwise Stratified Association

Associations of ASVs between oral and pancreatic tissues or intestinal site samples were investigated using two different types of statistical tests that account for pairing and within-subject correlation. For both tests, rarefied features with a relative abundance less than or equal to 0.01 (≤1%) were set to zero (i.e., set to absence), and only ASVs for which less than 95% of the samples exhibited a relative abundance of zero were tested.

The first test (Kappa) evaluated general concordance in the presence or absence of a given ASV in each subject. Specifically, for each subject, samples were divided into two groups: 1) oral samples, and 2) pancreatic tissue or intestinal site samples. The target ASV was denoted as present in a group if at least one of the samples had a relative abundance value larger than zero or denoted as absent if all relative abundance values in the group were zero. This reformatted data thus resulted in two values for each subject: (1) whether or not the target ASV was present in any of the oral samples from 4 oral sampling sites and (2) whether or not the target ASV was present in any of the pancreatic tissue or intestinal sites for that subject. Based on these values, Cohen’s Kappa concordance statistic was computed [27] utilizing the R package ‘irr’, and a one-sided test based on Kappa’s large sample standard deviation was conducted to examine whether there is a significant agreement in presence or absence between oral and pancreatic tissues or intestinal site samples for a given ASV.

Additionally, a test for Pairwise Stratified Association (PASTA) was performed to identify those ASVs (when present) which exhibit consistent patterns in relative abundance between oral and pancreatic tissues or intestinal site samples, after adjusting for disease status (represented by ICD10 codes) and within-subject correlations. PASTA test was based on a Bayesian regression model to obtain Markov–Chain Monte Carlo estimates of abundance among strata, to calculate a correlation statistic, and to conduct a formal test based on its posterior distribution. Samples were categorized by using four ICD10 codes ‘C24.*’, ‘C25.*’, ‘K86.2’ and ‘other’ (encodes other gastrointestinal conditions) or by three ICD10 codes ‘C24.*’, ‘C25.*’ and ‘other’ (includes K86.2 and other gastrointestinal conditions) to focus on neoplasms versus other conditions (Table 1). In contrast to tests based on Kappa statistics (which tests the concordance in presence or absence of a given ASV between groups), PASTA tests examine whether the differences in mean relative abundance across groups (e.g., disease status) are preserved between body sites (e.g., oral versus pancreatic or intestinal sites). An ASV was defined to exhibit a consistent PASTA pattern, if either one of the three possible quantities was associated between oral and pancreatic tissues or intestinal site samples: (1) The probability of absence (*p*), which denotes the probability of observing a relative abundance of zero, (2) The non-zero mean relative abundance (ω), which denotes mean relative abundance among those samples in which ASV is present; and (3) The mean relative abundance across all samples (μ). Further information about the data model, approach, and performance of the PASTA test has been published in detail [28] and briefly summarized in Supplemental Material 2.

**Table 1.** Characteristics of subjects analyzed (n = 52)

Characteristic	Mean (SD)
Age (years)	64.1 (12.5)
Body mass index (kg/m^2^)	26.3 (4.9), n = 51
	**n (%)**
Sex	
Male	24 (46)
Female	28 (54)
Race	
Caucasian	49 (94)
Black	1 (2)
Asian	2 (4)
Smoking status	
Ever smoker	32 (61.5)
Nonsmoker	19 (36.5)
Missing	1 (2)
Chemotherapy	
Never	37 (71.2)
Prior to past 6 months	5 (9.6)
In past 6 months	6 (11.5)
Missing	4 (7.7)
ICD-10 codes	
C24.0 (extrahepatic cholangiocarcinoma)	3 (5.8)
C25.0 – C25.9, C24.1 (pancreatic cancer and periampullary cancer)	29 (55.8)
K86.0 – K86.3 (other pancreatic conditions)	15 (28.8)
Other gastrointestinal conditions	5 (9.6)
Oral health	
Periodontal disease	4 (7.69) ^a^
Gum disease	5 (9.62) ^a^
Missing	12 (23.1)

SD = standard deviation. ^a^ 30 subjects with no periodontal or gum disease; 1

subject had both periodontal disease and gum disease.

Both Kappa and PASTA analyses were repeated by selecting only saliva samples at the oral site because all studies examining the association of the oral microbiome with pancreatic cancer risk [13–16] collected saliva samples.

#### ASV co-abundance groups

Microbial co-abundance analyses were conducted to elucidate the distinct strain-level cluster patterns among microbiome members. ASVs shared by more than 10% of the buccal, saliva, duodenum, and pancreatic tumor samples were considered prevalent ASVs. Correlations between these prevalent ASVs within each sampling site were calculated using the SparCC method (Sparse Correlations for Compositional data) [29]. The statistical significance of these correlation coefficients was assessed using a bootstrap procedure and then converted into a correlation distance matrix. Next, the Ward clustering algorithm, a top-down clustering approach, was used to cluster ASVs within each sampling site into co-abundance groups. Starting from the top of the Ward clustering tree, permutational MANOVA (9999 permutations, p < 0.001) was used to sequentially test whether any two branches of the tree were significantly different [30]. ASVs within the same co-abundance group increased or decreased in abundance together.

#### ASV co-abundance network

Bacterial co-abundance networks illustrate how groups of ASVs occupy different niches of the microbial ecosystem. The co-abundance networks were visualized as force-directed network plots using Python package NetworkX (version 2.2) with the spring layout of the Fruchterman-Reingold algorithm (k = 0.15) [31] for buccal, saliva, duodenum, and pancreatic tumor samples. Only ASVs with an absolute SparCC correlation value greater than 0.1 and a p-value less than 0.05 were plotted using force-directed algorithms [32]. Further details regarding methods used to visualize ASV co-abundance networks at different sampling sites are described in Supplemental Material 2.

## Results

### Population and sample characteristics

The present analysis included 52 subjects (Table 1). These subjects were between 31 and 86 years old, and contributed a total 316 sample (52 tongue swab, 46 buccal swab, 35 supragingival swab, 48 saliva samples, 22 duodenum tissue, 34 jejunum swab, and 19 bile duct swab samples, as well as 21 pancreatic ducts, 6 normal pancreatic tissues and 33 pancreatic tumor samples). Based on the pathology records, ICD10 codes were assigned to each subject: 24 (46%) subjects had pancreatic cancer, 8 (15%) subjects had periampullary adenocarcinoma and 4 (8%) subjects had extrahepatic cholangiocarcinoma, 11 (21%) subjects had other pancreatic conditions, and the remaining 5 (10%) had other gastrointestinal conditions. Sixty-two percent of these subjects were ever smokers and 87% received antibiotic days prior to surgery (after providing oral specimens). Five (10%) subjects had periodontal or gum disease.

A total of 4077 unique Amplicon Sequence Variants (ASVs) were identified across oral samples, and 4304 ASVs were identified across pancreatic tissue or intestinal tissue and swab samples via DADA2. ASVs are also referred to as ‘features’ in QIIME2 processing and can be roughly understood as unique identifiers reaching up to the levels of bacterial strains or a group of highly similar strains.

### Microbiome communities at different sites

**Figure 1.**
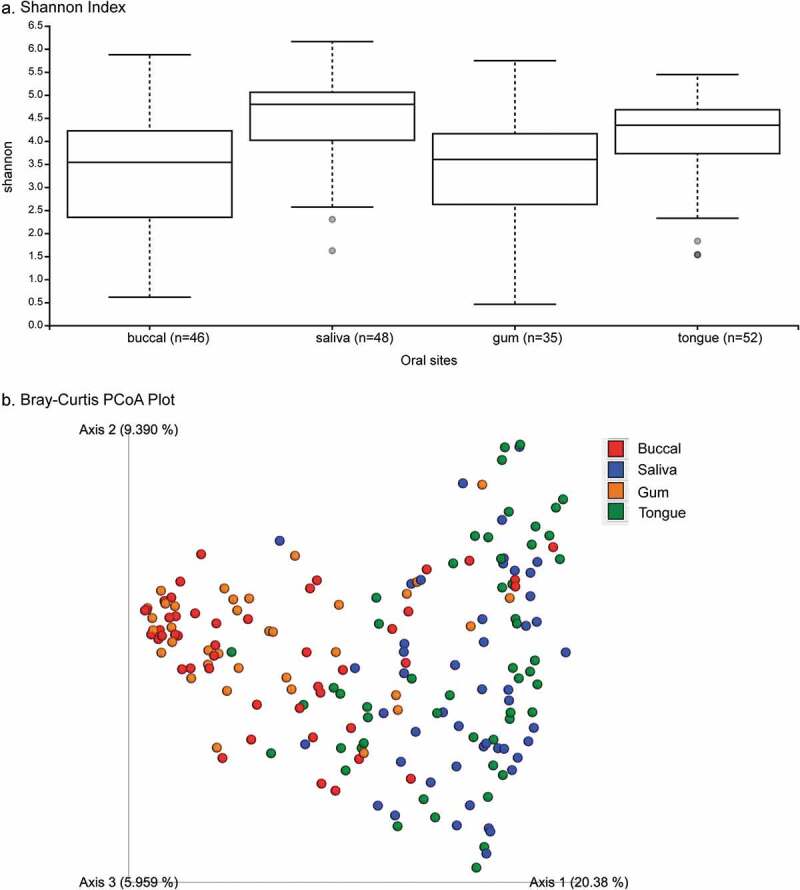
Alpha- and beta-diversity of oral microbiome among patients with pancreatic cancer and other gastrointestinal disease. Legends: Comparative alpha diversity (a. Shannon Index) and beta diversity (b. Bray–Curtis PCoA plot) analyses of bacterial communities in buccal, saliva, gum, and tongue sites

**Figure 2.**
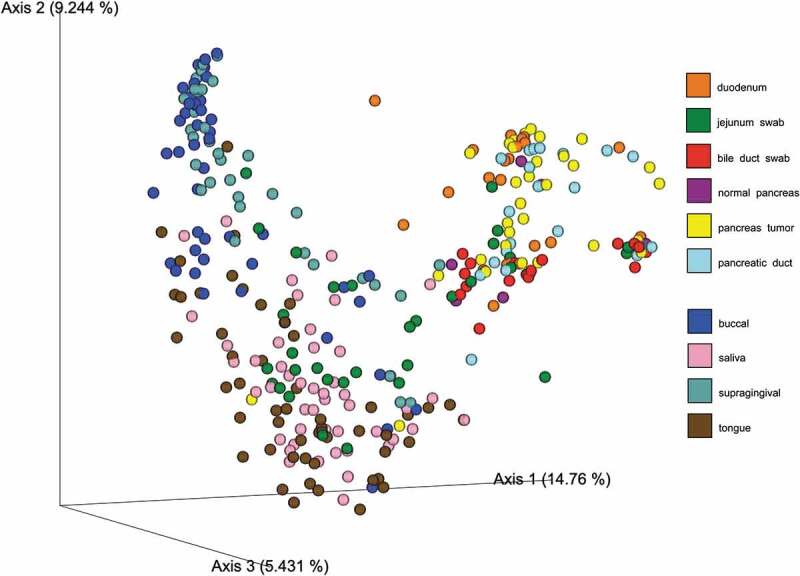
Bray–Curtis dissimilarity index plot across all sampling sites. Legends: Bray–Curtis PCoA plot showing the relatedness of microbial communities among the 52 subjects across all sampling sites (i.e., duodenum, jejunum swab, bile duct swab, normal pancreas, pancreas tumor, pancreatic duct, buccal saliva, gum, and tongue sites)

Based on the Shannon index (alpha-diversity measure), saliva and tongue samples were more diverse (median Shannon index >4) than buccal and supragingival samples (Figure 1(a)). The coordination of the Bray-Curtis PCoA plot (beta-diversity measure) also revealed two clustering groups. Specifically, bacterial communities from tongue and saliva samples clustered together, while those from buccal and supragingival samples formed another cluster (Figure 1(b)). Bacterial communities in pancreatic tissue and intestinal tissue or surfaces in this population have been previously described [17]. Briefly, alpha and beta-diversity analyses did not show any visually apparent clustering by sites (i.e., duodenum tissue, jejunum swab, bile duct swab, pancreatic duct, and pancreatic tissue samples). However, there were clear separations between oral bacterial communities and bacterial communities from duodenum tissue, bile duct swab, pancreatic duct, and pancreatic tissue samples; only bacterial communities from jejunum swabs clustered with oral bacterial communities (Figure 2)

PERMANOVA tests indicated that all samples were significantly different from each other, except those bacterial communities from buccal samples were not different from supragingival samples (q = 0.14) or saliva samples (q = 0.09), and the pancreatic duct samples were not different from pancreatic tumor samples (q = 0.28). (S1 Table)

### Shared ASVs and taxonomy

**Figure 3.**
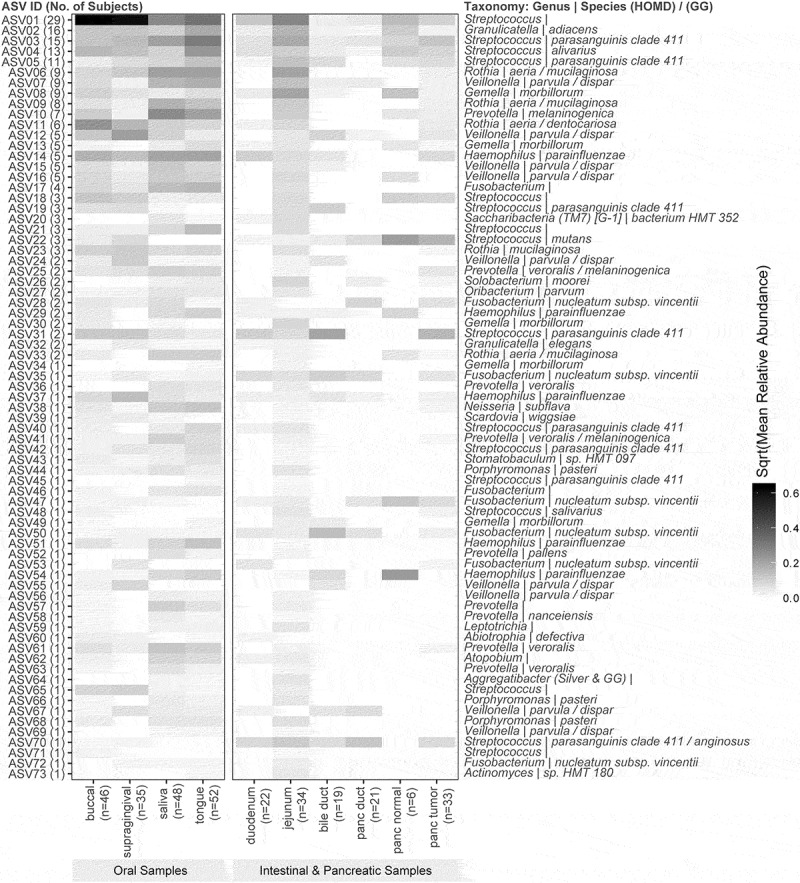
Shared ASVs (number of subjects) and Relative Abundance by Sampling Sites. Legends: On the left panel of the heatmap, each ASV is labeled with an ASV ID and the number of subjects who have the ASV present in both their oral and intestinal and pancreatic samples. Taxonomic annotations of these shared ASV are also provided on the right panel of the heatmap

A total of 73 ASVs were common between oral (any site) and intestinal or pancreatic samples in at least one subject (Figure 3; S2 Table). Taxonomic annotations of these shared ASV indicate that *Streptococcus, Veillonella, Prevotella, Fusobacterium, Gemella, Haemophilus*, and *Rothia* are the top seven frequently shared genera (in descending order) between oral and gut or pancreatic samples. The top seven most frequently shared species include *Veillonella parvula, Streptococcus parasanguinis clade 411, Fusobacterium nucleatum subsp. vincentii, Gemella morbillorum, Haemophilus parainfluenzae, Prevotella veroralis*, and *Rothia aeria*.

### Concordance (Kappa) and Pairwise Stratified Association (PASTA) between oral and pancreatic tissue or intestinal samples

For both Kappa and PASTA tests, a total of 50 ASVs for which less than 95% of the samples exhibited a relative abundance of zero were tested. Based on the Kappa statistics, four ASVs showed significant concordance with regards to the probability of presence or absence between any oral sample and pancreatic tissue or intestinal samples after adjusting for multiple testing. The taxonomy of these ASVs (same assigned taxa in all reference databases unless otherwise noted) are: *Fusobacterium nucleatum subsp. vincentii, Rothia mucilaginosa (GG), Gemella morbillorum*, and *Rothia aeria (HOMD)/mucilaginosa (GG)*. However, the estimated probability to be present in both oral and pancreatic tissue or intestinal samples were low (ranging from 3.8% to 17%) for these four ASVs (Table 2). When analyzing only saliva samples, total of seven ASVs showed significant concordance between saliva and pancreatic tissue or intestinal samples, including *F. nucleatum subsp. vincentii* (two ASVs), *Rothia mucilaginosa (GG), and Gemella morbillorum, Saccharibacteria_(TM7)_[G-1] bacterium_HMT_352, Oribacterium parvum*, genus *Streptococcus, and* genus *Fusobacterium*. The estimated probability to be present in both saliva and pancreatic tissue or intestinal samples ranged widely from 2.1% to 56% for these seven ASVs (Table 3).

**Table 2.** Four ASVs that showed significant agreement* with regards to the probabilities of presence or absence (Kappa statistics) between any oral site and any pancreatic tissue or intestinal samples

ASV ID^b^	Kappa (SD)	q-value	Agree obs	Prop.pp	Prop.pa	Prop.ap	Prop.aa	Genus^a^	Species (HOMD)^a^
ASV28	0.321 (0.102)	0.020	0.865	0.038	0.135	0.000	0.827	*Fusobacterium*	*nucleatum subsp. vincentii*
ASV23	0.397 (0.125)	0.020	0.865	0.058	0.115	0.019	0.808	*Rothia*	*mucilaginosa (GG)*
ASV13	0.353 (0.133)	0.048	0.788	0.096	0.154	0.058	0.692	*Gemella*	*morbillorum*
ASV06	0.316 (0.118)	0.048	0.673	0.173	0.288	0.038	0.500	*Rothia*	*aeria/mucilaginosa GG)*

Agree obs = observed agreement; ASV = Amplicon Sequence Variants; SD = standard deviation; q-value = p-value adjusted for multiple testing via the false discovery rate method; Prop. pp = probability of an ASV to be present in both oral and any pancreatic tissue or intestinal samples; Prop. pa = probability of an ASV to be present in oral samples but absent in pancreatic or intestinal samples; Prop. ap = probability of an ASV to be absent in oral samples but present in pancreatic or intestinal samples; Prop. aa = probability of an ASV to be absent in both oral and any pancreatic tissue or intestinal samples.

*Significant agreement/concordance can be reached if probability of an ASV to be present in both oral and any pancreatic tissue or intestinal samples (Prop. pp) or probability of an ASV to be absent in both oral and any pancreatic tissue or intestinal sample (Prop. aa) is high.

^a^TAXONOMIC annotations of the ASVs are from HOMD database unless otherwise noted. GG = green gene database.

^b^ASV ID used in Figure 1 left panel.

**Table 3.** Seven ASVs that showed significant agreement* with regards to the probabilities of presence or absence (Kappa statistics) between saliva and any pancreatic tissue or intestinal samples

ASV ID^b^	Kappa (SD)	q-value	Agree obs	Prop.pp	Prop.pa	Prop.ap	Prop.aa	Genus^a^	Species (HOMD)^a^
ASV28	0.406 (0.116)	0.004	0.896	0.042	0.104	0.000	0.854	*Fusobacterium*	*nucleatum subsp. vincentii*
ASV23	0.492 (0.137)	0.004	0.896	0.063	0.083	0.021	0.833	*Rothia*	*mucilaginosa (GG)*
ASV20	0.431 (0.142)	0.013	0.875	0.063	0.083	0.042	0.813	*Saccharibacteria_(TM7)_[G-1]*	*bacterium_HMT_352*
ASV27	0.357 (0.111)	0.009	0.875	0.042	0.125	0.000	0.833	*Oribacterium*	*parvum*
ASV01	0.344 (0.119)	0.016	0.708	0.563	0.271	0.021	0.146	*Streptococcus*	
ASV47	0.309 (0.104)	0.014	0.917	0.021	0.000	0.083	0.896	*Fusobacterium*	*nucleatum subsp. vincentii*
ASV17	0.284 (0.101)	0.017	0.729	0.083	0.271	0.000	0.646	*Fusobacterium*	

Agree obs = observed agreement; ASV = Amplicon Sequence Variants; SD = standard deviation; q-value = p-value adjusted for multiple testing via the false discovery rate method; Prop. pp = probability of an ASV to be present in both oral and any pancreatic tissue or intestinal samples; Prop. pa = probability of an ASV to be present in oral samples but absent in pancreatic or intestinal samples; Prop. ap = probability of an ASV to be absent in oral samples but present in pancreatic or intestinal samples; Prop. aa = probability of an ASV to be absent in both oral and any pancreatic tissue or intestinal samples.

*Significant agreement/concordance can be reached if probability of an ASV to be present in both oral and any pancreatic tissue or intestinal samples (Prop. pp), or probability of an ASV to be absent in both oral and any pancreatic tissue or intestinal sample (Prop. aa) is high.

^a^TAXONOMIC annotations of the ASVs are from HOMD database unless otherwise noted. GG = green gene database.

^b^SV ID used in Figure 1 left panel.

The PASTA test identified two ASVs (ASV13 and ASV21), *Gemella morbillorum* and genus *Streptococcus*, that showed consistent presence or absence patterns between oral and intestinal or pancreatic samples, after adjusting for within-subject correlation and disease status. Detailed analyses are reported in the Supplemental Material 3. The ASVs corresponding to *Gemella morbillorum* (ASV13) and *Fusobacterium nucleatum subsp. vincentii* (ASV28) showed marginally significant associations with respect to *p* only when only saliva samples were used to compare to pancreatic tissue or intestinal samples.

### Patterns of co-abundance between ASVs per body site

A co-abundance network diagram and clustering tree were graphed for the prevalent ASVs in the saliva (S1 Figure, panel a and b), buccal (S2 Figure, panel a and b), duodenum (S3 Figure, panel a and b), jejunum (S4 Figure, panel a and b), and pancreatic tumor samples (S5 Figure, panel a and b), respectively.

The Ward clustering algorithm identified six and five co-abundance clusters in saliva and buccal samples, respectively (S1 and S2 Figures). In buccal samples (S2 Figure), the cluster dominated by *Veillonella parvula* (Red and Green) was inversely correlated with the co-abundance cluster that mostly contained *Fusobacterium nucleatum subsp. vincentii, Haemophilus parainfluenzae*, and *Capnocytophaga gingivalis* (Orange). In saliva samples (S1 Figure panel b), the same *Capnocytophaga gingivalis* ASV, which was also in buccal samples, was clustered in a co-abundance group with many *Fusobacterium nucleatum subsp. vincentii* and *Streptococcus* ASVs (Grey). Moreover, the co-abundance cluster that contained only *Fusobacterium, Neisseria*, and *Haemophilus parainfluenzae* (Green) was more positively correlated with a cluster dominated by *Haemophilus* ASVs and *Streptococcus* ASVs (Yellow), and more inversely correlated with a cluster of *Veillonella parvula* ASVs and *Prevotella veroralis* ASVs (Red). The five ASVs identified by the PASTA tests belonged to the grey cluster (ASV28, ASV21, ASV13, and ASV19) and the green cluster (ASV67) in buccal samples. They belonged to the yellow cluster (ASV21 and ASV19), pink cluster (ASV 13 and ASV 67), and grey cluster (ASV28) in saliva samples.

There were 3, 8, and 4 co-abundance clusters in the duodenum tissue, jejunum swab, and pancreatic tumor tissue samples, respectively (S3, S4, and S5 Figures). All five ASVs identified by the PASTA tests were shown in the jejunum swab clusters – three ASVs (ASV13, ASV28, ASV67) belonged to the purple cluster, one (ASV19) belonged to the yellow cluster, and one (ASV21) belonged to the pink cluster. One duodenum cluster was dominated by *Fusobacterium nucleatum subsp. vincentii* ASVs and *Klebsiella pneumoniae* ASVs (Red), and was inversely correlated to a cluster (Green) that contained *Ralstonia, Bacteroides, Lachnospiraceae, Saccharibacteria, Brevundimonas*, and *Sphingomonas* ASVs. In the pancreatic tumor samples, one cluster was dominated by *Fusobacterium nucleatum subsp. vincentii, Klebsiella pneumoniae, Campylobacter rectus, Dialister pneumosintes, Prevotella nigrescens*, and *Parvimonas* micra ASVs (Red), which contains bacteria that were identified as ‘the orange complex’ – the transitional population between health and severe periodontal disease [33]. Only two (ASV13 and ASV67) of the five ASVs identified by the PASTA tests were shown in the clusters of ASVs in duodenum samples. Both (*Gemella morbillorum* and *Veillonella parvula*) belonged to the grey cluster. Two ASVs (ASV13: *Gemella morbillorum* and ASV67: *Veillonella| arvula*) consistently belonged to the same cluster in saliva, duodenum tissue, and jejunum swab samples. None of the five ASVs were plotted in pancreatic tumor samples’ ASV clusters (due to low or insignificant correlations with other ASVs) although two (ASV13 and ASV28) were present in the pancreatic tumor samples (S1 Table).

## Discussions

To our knowledge, this is the first study to date to characterize oral bacterial microbiome communities at four oral sites (tongue, buccal, supragingival, and saliva) and examined their correlations with the microbiome in the pancreatic tissue or intestinal samples using several complementary analyses. Due to a small number of pancreatic tissue and intestinal samples, our analyses combined all available samples to increase statistical power. In this study, we identified many ASVs that were shared between oral and pancreatic or intestinal samples among patients with pancreatic cancer and other gastrointestinal diseases, even though pancreatic and intestinal samples had a much lower number and abundance of ASVs compared to oral samples. We also identified a number of bacteria that were correlated in oral samples and pancreatic tissue samples and may have relevance in the underlying disease.

Our results expand the original finding of the Human Microbiome Project of oral biogeography in healthy subjects [34]. When comparing oral (all sampling site combined) to intestinal or pancreatic samples, four ASVs showed significant concordance (Kappa statistics) and five ASVs exhibited significant or marginally significant associations (PASTA) with regards to the probabilities of absence or presence. We do not have sufficient statistical power to examine which oral site might be the ‘best’ oral sampling site; however, we did compare saliva to intestinal sites as saliva is often the collection of choice in observational cohort studies. Overall, the concordance of the ASVs (when present) between any oral site and pancreatic or intestinal sites was low, suggesting that while oral bacteria are likely migrating to the gastrointestinal sites, oral samples may now be used to measure the bacteria in the pancreas directly. When analyzing overall microbial communities at different sampling sites, the microbial co-abundance analyses illustrate the distinct strain-level cluster patterns among microbiome members in buccal, saliva, duodenum, and pancreatic tumor samples. Our results provide initial insights for future studies to begin to uncover and define the co-abundance of specific oral microbiome communities and to explore their potential roles in monitoring the process of pancreatic carcinogenesis or disease progressions.

Emerging research has focussed on profiling oral microbiome as potential biomarkers for disease phenotypes in epidemiological studies [35] because they are noninvasive, and oral microbiome profiles have shown relative intraindividual stability over time and clear interindividual differences [36,37]. Although many observational studies have shown that specific oral microbiota in oral cavities and in the fecal samples are associated with oral, head and neck, lung, colorectal, and pancreatic cancer risks [7,8,38], data on the associations between oral and tissue microbiome profiles are very limited. To our knowledge, there is only one prior study that examined the microbial profiles that were shared between the oral cavity and tissue samples [39]. Their analyses have focussed on 17 OTUs that were detected in 37% of both tissue samples (CRC and polyp) and oral swabs, and identified two tumor-associated bacterial co-abundance clusters. Specifically, one cluster is comprised of oral pathogens previously linked with late colonization of oral biofilms and with human diseases including CRC (e.g., *Fusobacterium nucleatum, Parvimonas micra, Peptostreptococcus stomatis*, and *Dialister pneumosintes*), and the other cluster is comprised of dominant bacteria in early dental biofilm formation including genera *Actinomyces, Haemophilus, Rothia, Streptococcus*, and *Veilonella* [39]. A letter to the editor reported that *Fusobacterium nucleatum* was detected in 8 (57.1%) of 14 CRC patients’ tumor and saliva samples, and identical strains were found in 75% of the tumor and saliva samples suggesting that *F. nucleatum* in colorectal tumor originates in the oral cavity [40]. In the present study, we identified 73 ASVs shared between oral and intestinal or pancreatic (Figure 2; S1 Table). In our study, only *F. nucleatum* was identified from the 1^st^ cluster of the work of Flemmer et al. [39], however, all except *Actinomyces* was found from the second cluster. We found that *F. nucleatum* was among the top three most frequently shared species based on the taxonomic annotations of the shared ASVs between oral and intestinal or pancreatic samples.

Our co-abundance analyses identified multiple clusters of ASVs in buccal, saliva, duodenum, jejunum swab, and pancreatic tumor samples. In buccal and saliva samples, *Capnocytophaga gingivalis* ASV was consistently found in the same co-abundance group with *Fusobacterium nucleatum subsp. vincentii* ASVs. Of the four clusters of ASVs in pancreatic tumor samples, one cluster with the largest number of ASVs comprised mostly the dominant bacteria in early dental biofilm formation or the genera also associated with relatively healthy tooth pockets [33], and the other three clusters each contain bacterial species that are associated with cancer risks such as *Gemella morbillorum* and *Fusobacterium nucleatum subsp. vincentii*, as well as species that are associated with periodontal disease or infections such as *Prevotella nigrescens, Campylobacter rectus, and Klebsiella pneumoniae* [41,42]. A recent study investigating the microbiome of intraductal papillary mucous neoplasms, lesions which can progress to pancreatic cancer, also demonstrated that the oral pathogen *F. nucleatum* co-occurred with 10 other species, including *Serratia marcescens, Parvimonas micra, Prevotella melaninogenica, Haemophilus parahaemolyticus, Streptococcus anginosus, Bergeyella sp*. HMT322, *Kluyvera ascorbata, Eikenella corrodens, Campylobacter concisus,* and *Campylobacter showae*, many of which are known oral species [43].

After adjusting for disease status, our PASTA analyses identified two specific bacterial species (*Gemella morbillorum and Fusobacterium nucleatum subsp. vincentii*) that showed consistent presence or absence patterns between oral and intestinal or pancreatic samples, which warrant further investigations on their associations to the pancreatic cancer risk or progression. A few studies had shown associations between these bacterial species and CRC risk. A large retrospective study found that the risk of CRC was significantly increased in patients with culture-confirmed bacteremia (presence of bacteria in the blood) from *Gemella morbillorum*, previously known as *Streptococcus morbillorum* (HR = 15.2; 95% CI = 1.54–150), and from *Fusobacterium nucleatum* (HR = 6.89; 95% CI = 1.70–27.9) [44]. A case-control study in a cohort of individuals undergoing screening colonoscopy found that both *Gemella morbillorum and F. nucleatum* (part of 21 species-level OTUs) were enriched in fecal samples from CRC patients compared to controls [38]. This study also showed strong co-abundance relationships between *Parvimonas micra, F. nucleatum, and Solobacterium moorei*. Another study analyzed the association of *Fusobacterium* species in pancreatic tumor with patient prognosis and showed significantly higher mortality (poorer prognosis) among pancreatic cancer patients with *Fusobacterium* species-positive tumors than those with *Fusobacterium* species-negative tumors (HR = 2.16; 95% CI = 1.12–3.91) [45].

A growing number of studies have investigated the relationship between the oral microbiome and pancreatic cancer risk using different methods and study designs, but the results have been inconsistent, and most studies had small sample sizes [13–16]. The largest study to date of the oral microbiome and pancreatic cancer capitalized on data collected on patients in two large prospective cohort studies to conduct a nested case-control study [13]. The results showed that presence (vs absence) of *P. gingivalis* and *Aggregatibacter actinomycetemcomitans* in saliva collected prior to cancer diagnosis was associated with a 60% and 120% increase in the risk of pancreatic cancer, respectively. In contrast, the presence (vs absence) of *Leptotrichia* in saliva was associated with a 13% decreased risk of pancreatic cancer [13]. None of the bacterial species or genus that were different in saliva samples comparing pancreatic cancer patients to healthy controls (in prior studies) showed significant concordance or PASTA associations between saliva and pancreatic tissue or intestinal samples in our analyses. The most recently published study evaluated the characteristics of the oral microbiota in patients with pancreatic ductal adenocarcinoma (PDAC), intraductal papillary mucinous neoplasms (IPMNs), and healthy controls [15]. This case-control study found no differences between patients with PDAC and healthy controls or between patients with PDAC and those with IPMNs, on measures of alpha diversity of the oral microbiota. PDAC patients had higher levels of Firmicutes and several related taxa (*Bacilli, Lactobacillales, Streptococcaceae, Streptococcus, Streptococcus thermophilus*), although, after adjustment for multiple testing, results remained significant at the phylum level only. In our data, an ASV corresponding to genus *Streptococcus* was the only ASV showing a high probability (56%) to be present in both saliva and pancreatic tissue or intestinal samples.

Our study has several strengths. We used ASVs from Illumina-scale amplicon data in all analyses without imposing the arbitrary dissimilarity thresholds that define molecular OTUs. ASVs capture all biological variation present in the data, and ASVs are consistently labeled with intrinsic biological meaning as a DNA sequence [46]. By analyzing ASVs, our analyses overcame the main challenge of 16S rRNA sequence taxonomic classification, that is selecting appropriate marker genes that contain sufficient heterogeneity in order to ensure accurate differentiation of target species [47]. Additionally, our study results can be reliably reproduced and validated in future studies. However, our study also had some limitations; our sample size was small and PASTA tests were likely underpowered. About 20% of our study population received chemotherapy and 87% received antibiotic days prior to surgery (after providing oral specimens), which may confound our findings. Moreover, we did not have samples from healthy controls and could not compare our findings with prior studies due to differences in methods.

## Conclusions

Taken together, the results of the present study suggest that members of oral, intestinal, and pancreatic bacterial microbiomes overlap but exhibit distinct co-abundance patterns in patients with pancreatic cancer and other gastrointestinal diseases. Our findings provide critical insights on microbial communities and species that are common in the oral cavity, intestinal, and pancreatic tissue samples among pancreatic cancer patients and other gastrointestinal diseases. Though due to the cross-sectional study design, we are unable to make any conclusion regarding their roles in disease progression or whether these bacterial species were colonizing or just passing through various body sites. Our findings should be validated in independent and adequately sized populations with appropriate controls. Moreover, growing evidence have shown that bacterial species may survive, decline, and adapt as interdependent functional groups responding to environmental changes [30,48,49]. Future studies should aim to uncover the co-abundance of specific microbial communities to explore their potential roles in the etiology of microbiota-driven carcinogenesis in prospective and longitudinal studies.

## Supplementary Material

Supplemental Material

## Data Availability

Sequence data have been deposited in Sequence Read Archive (SRA): https://www.ncbi.nlm.nih.gov/sra/PRJNA558364
