# Supplementary material for: Comparisons of oral, intestinal, and pancreatic bacterial microbiomes in patients with pancreatic cancer and other gastrointestinal diseases
Source: J Oral Microbiol. 2021 Feb 14;13(1):1887680. doi: 10.1080/20002297.2021.1887680 (PMC7889162; doi:10.1080/20002297.2021.1887680)
Supplement: Supplemental Material [file ZJOM_A_1887680_SM1924.zip › Supplementary files/S1 Table rv1.docx]

**Supplemental Table 1.** PERMANOVA pairwise comparison results of beta-diversity measures between sampling sites.

| **Group 1** | **Group 2** | **Sample size** | **Permutations** | **pseudo-F** | **p-value** | **q-value*** |
| --- | --- | --- | --- | --- | --- | --- |
| Bile duct swab | duodenum | 41 | 999 | 21.8732518 | 0.001 | 0.00128571 |
| Bile duct swab | jejunum swab | 53 | 999 | 3.28641728 | 0.008 | 0.00929032 |
| Bile duct swab | panc duct | 40 | 999 | 9.51120681 | 0.001 | 0.00128571 |
| Bile duct swab | panc tumor | 50 | 999 | 12.7326542 | 0.001 | 0.00128571 |
| Bile duct swab | buccal mucosa | 65 | 999 | 13.842031 | 0.001 | 0.00128571 |
| Bile duct swab | saliva | 67 | 999 | 17.388981 | 0.001 | 0.00128571 |
| Bile duct swab | supragingival plaque | 54 | 999 | 10.5651247 | 0.001 | 0.00128571 |
| Bile duct swab | tongue | 71 | 999 | 17.0755316 | 0.001 | 0.00128571 |
| duodenum | jejunum swab | 56 | 999 | 23.735941 | 0.001 | 0.00128571 |
| duodenum | panc duct | 43 | 999 | 7.68258035 | 0.001 | 0.00128571 |
| duodenum | panc tumor | 53 | 999 | 6.53111612 | 0.001 | 0.00128571 |
| duodenum | buccal mucosa | 68 | 999 | 61.5226052 | 0.001 | 0.00128571 |
| duodenum | saliva | 70 | 999 | 67.2401392 | 0.001 | 0.00128571 |
| duodenum | supragingival plaque | 57 | 999 | 52.7399691 | 0.001 | 0.00128571 |
| duodenum | tongue | 74 | 999 | 76.7908222 | 0.001 | 0.00128571 |
| jejunum swab | panc duct | 55 | 999 | 10.970225 | 0.001 | 0.00128571 |
| jejunum swab | panc tumor | 65 | 999 | 14.64246 | 0.001 | 0.00128571 |
| jejunum swab | buccal mucosa | 80 | 999 | 9.67028549 | 0.001 | 0.00128571 |
| jejunum swab | saliva | 82 | 999 | 12.1459049 | 0.001 | 0.00128571 |
| jejunum swab | supragingival plaque | 69 | 999 | 8.5099804 | 0.001 | 0.00128571 |
| jejunum swab | tongue | 86 | 999 | 11.9476061 | 0.001 | 0.00128571 |
| panc duct | panc tumor | 52 | 999 | 1.14352074 | 0.28 | 0.28 |
| panc duct | buccal mucosa | 67 | 999 | 35.1388425 | 0.001 | 0.00128571 |
| panc duct | saliva | 69 | 999 | 39.3743388 | 0.001 | 0.00128571 |
| panc duct | supragingival plaque | 56 | 999 | 29.675237 | 0.001 | 0.00128571 |
| panc duct | tongue | 73 | 999 | 44.7790423 | 0.001 | 0.00128571 |
| panc tumor | buccal mucosa | 77 | 999 | 44.8528388 | 0.001 | 0.00128571 |
| panc tumor | saliva | 79 | 999 | 49.8322844 | 0.001 | 0.00128571 |
| panc tumor | supragingival plaque | 66 | 999 | 37.3665979 | 0.001 | 0.00128571 |
| panc tumor | tongue | 83 | 999 | 56.5020865 | 0.001 | 0.00128571 |
| buccal mucosa | saliva | 94 | 999 | 1.65231133 | 0.086 | 0.09105882 |
| buccal mucosa | supragingival plaque | 81 | 999 | 1.4367544 | 0.136 | 0.13988571 |
| buccal mucosa | tongue | 98 | 999 | 2.41520065 | 0.011 | 0.012375 |
| saliva | supragingival plaque | 83 | 999 | 3.41093077 | 0.003 | 0.0036 |
| saliva | tongue | 100 | 999 | 2.42743864 | 0.021 | 0.02290909 |
| supragingival plaque | tongue | 87 | 999 | 3.57415991 | 0.003 | 0.0036 |

panc = pancreatic. *A false discovery rate (FDR) adjusted p-value (or q-value) less than 0.05 was considered significant difference in beta-diversity measures between oral sites.
