# Supplementary material for: Comparisons of oral, intestinal, and pancreatic bacterial microbiomes in patients with pancreatic cancer and other gastrointestinal diseases
Source: J Oral Microbiol. 2021 Feb 14;13(1):1887680. doi: 10.1080/20002297.2021.1887680 (PMC7889162; doi:10.1080/20002297.2021.1887680)
Supplement: Supplemental Material [file ZJOM_A_1887680_SM1924.zip › Supplementary files/S1_to_S5 Figures Combined_rv1.pdf]

S1a. Saliva co-abundance network

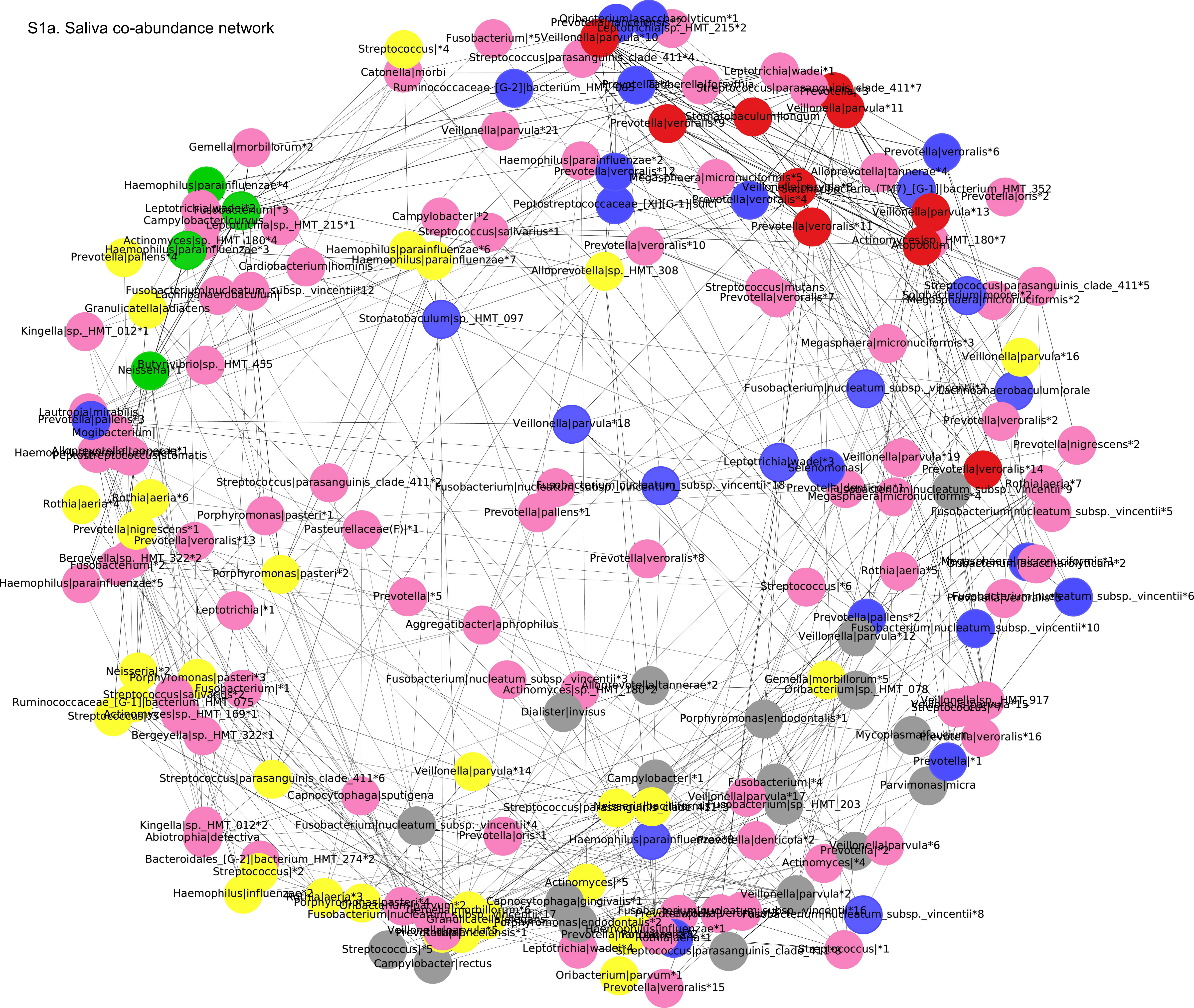

S1b.

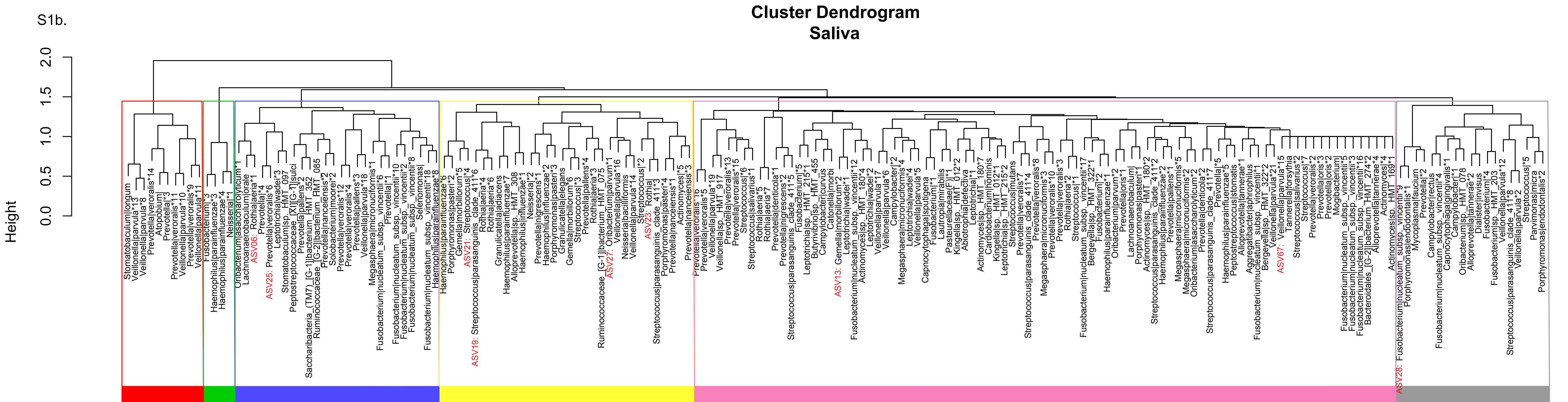
Cluster Dendrogram
Saliva
Height
0.0
0.5
1.0
1.5
2.0
ASV06
ASV25
ASV21
ASV19
ASV27
ASV23
ASV13
ASV67
ASV28

S2a. Buccal co-abundance network

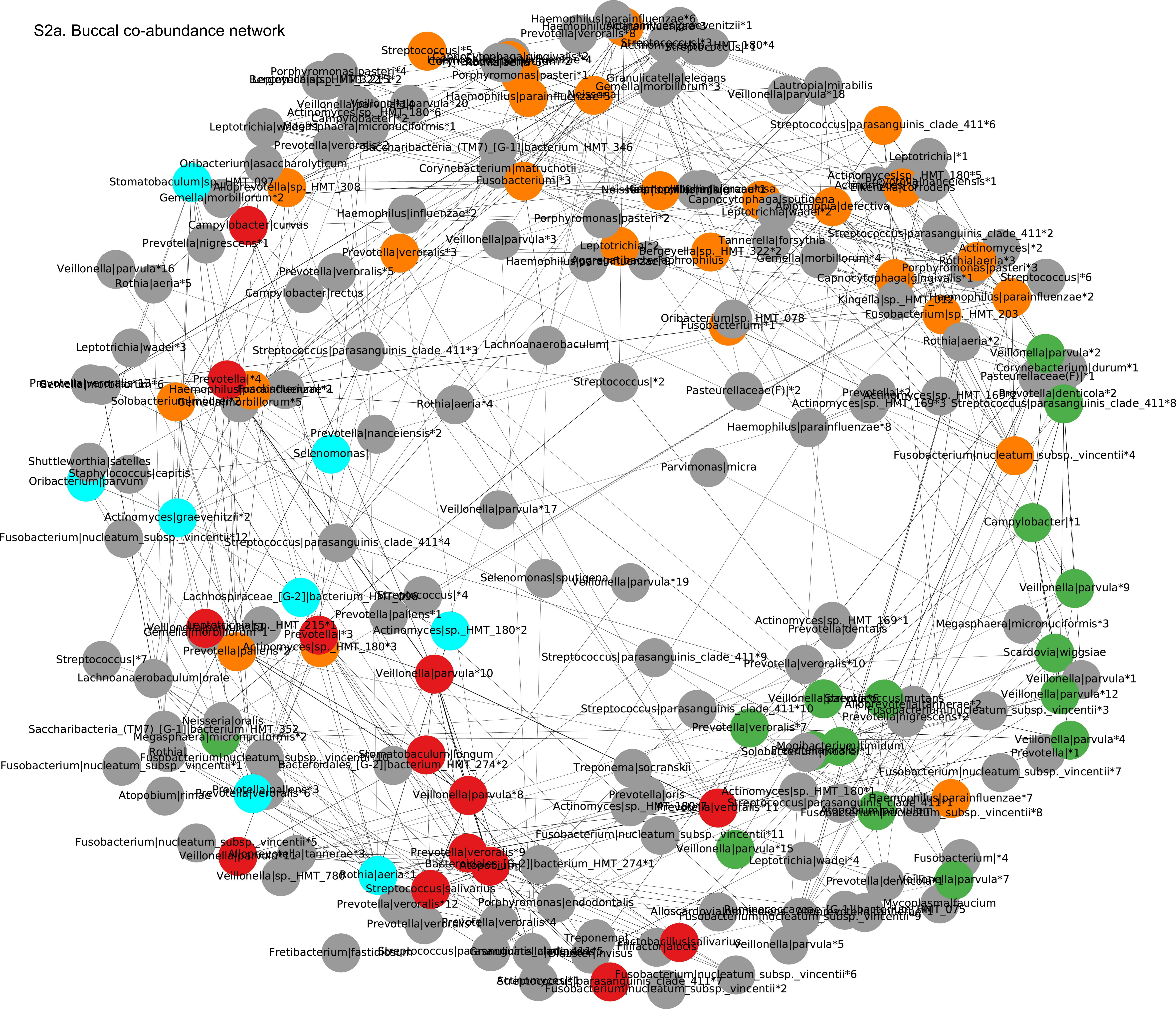

S2b.

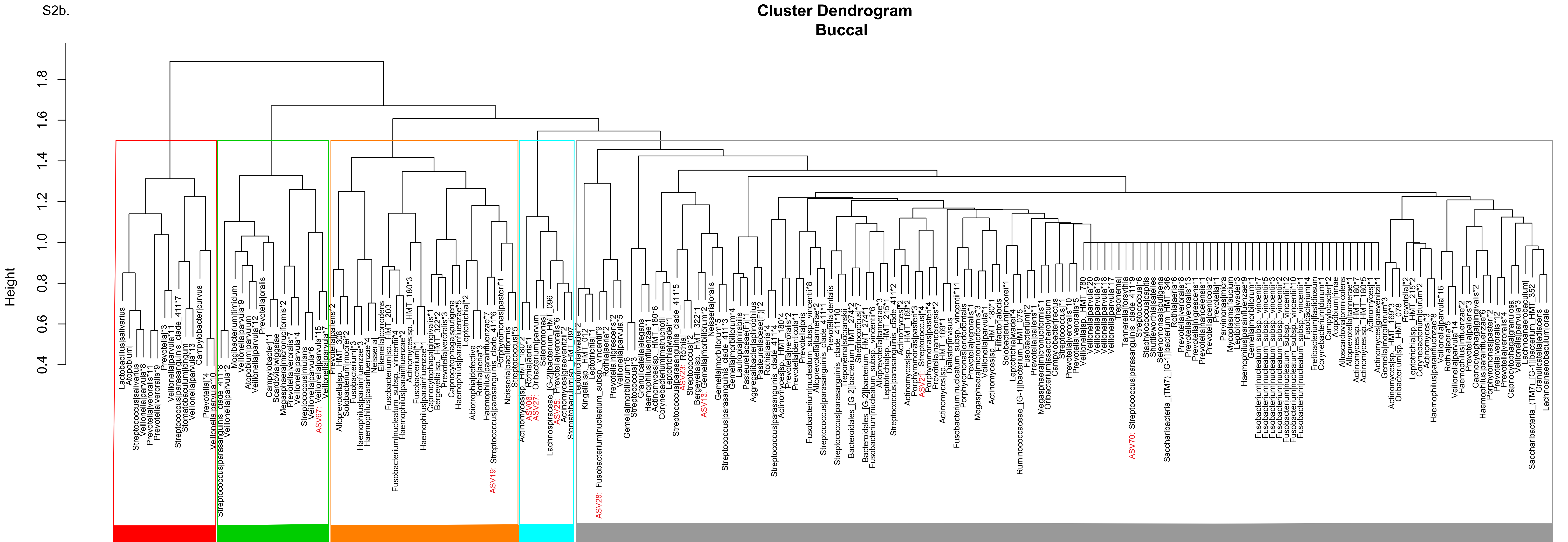

S3a. Duodenum co-abundance network

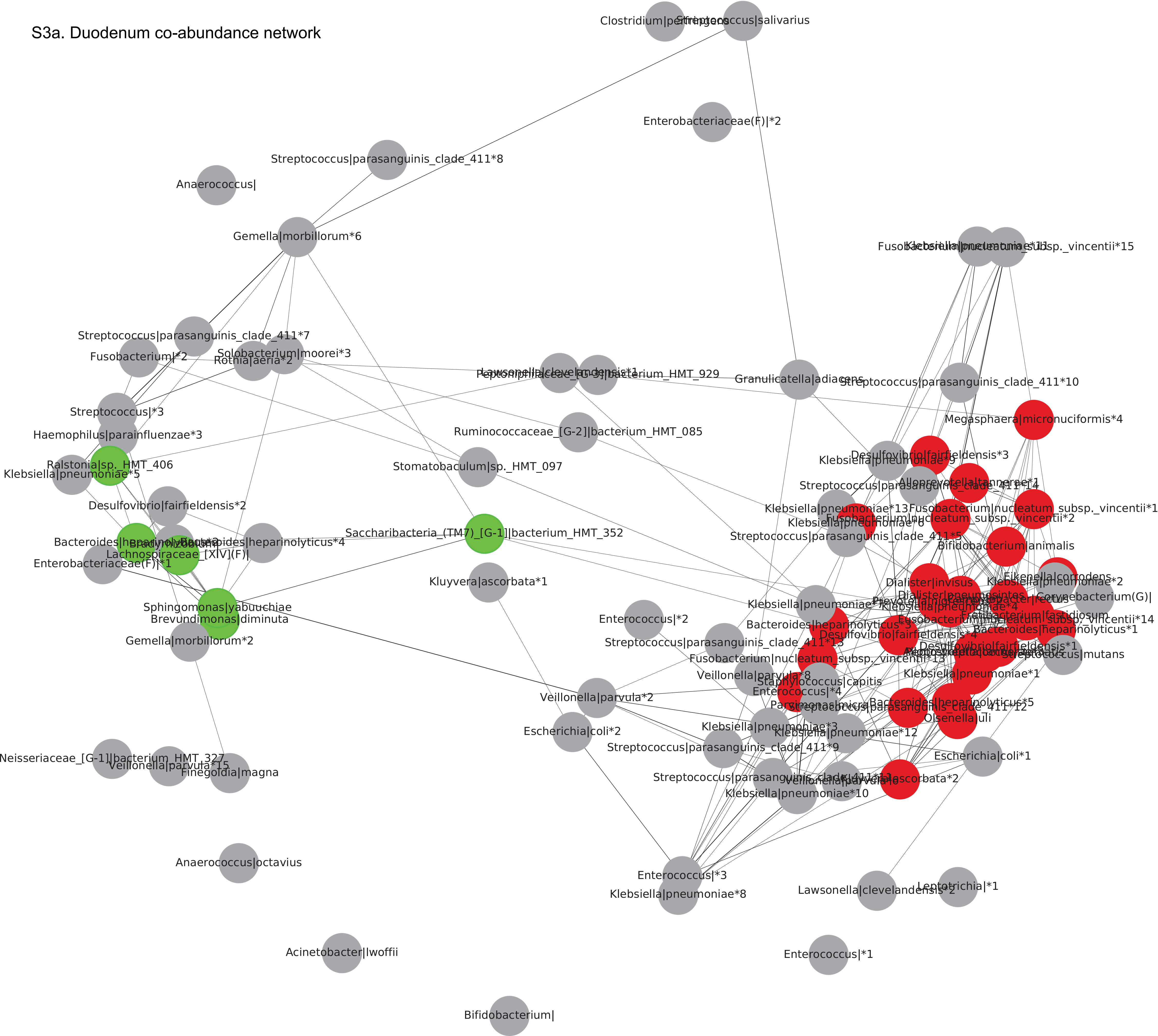

S3b.

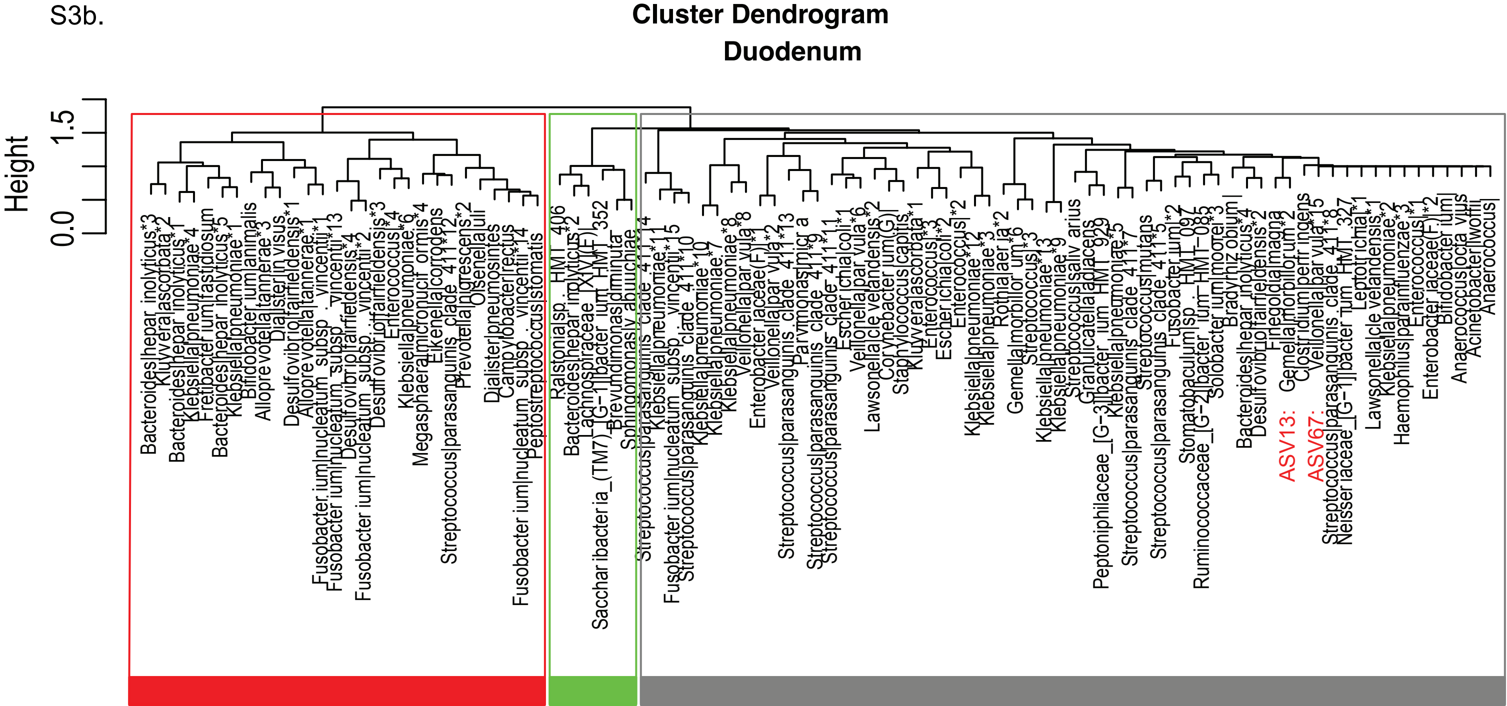

S4a. Jejunums co-abundance network

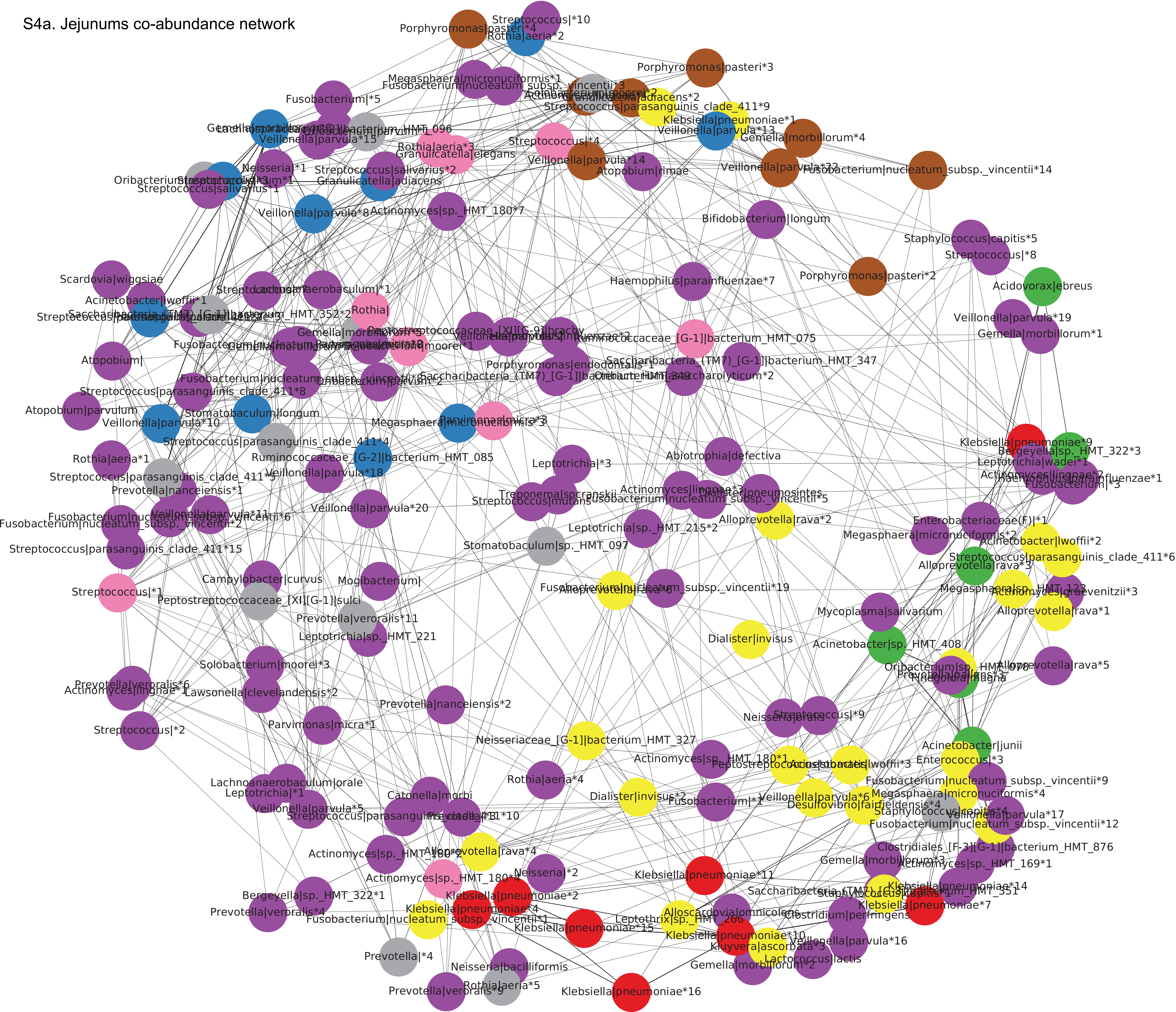

S4b.

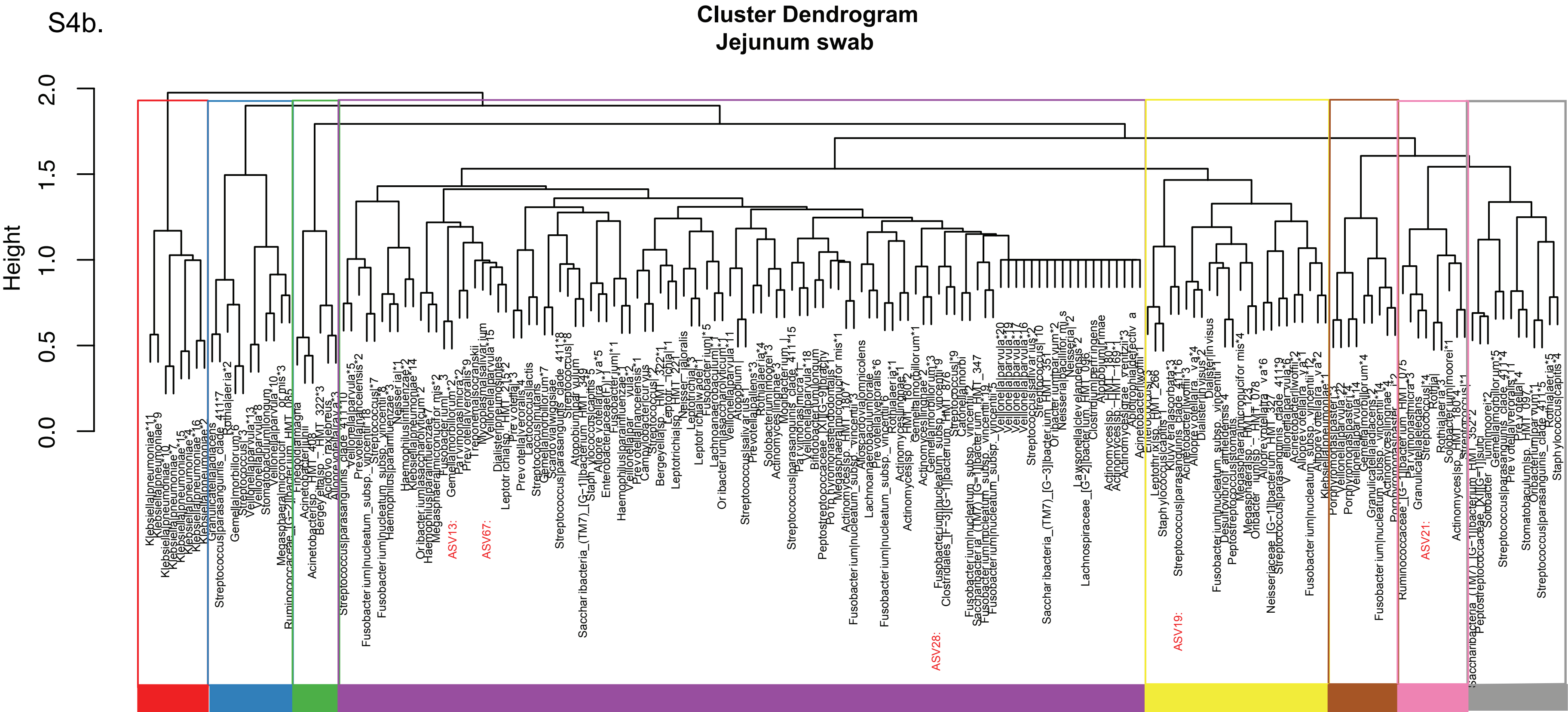
Cluster Dendrogram
Jejunum swab
Height
0.0
0.5
1.0
1.5
2.0
ASV13:
ASV67:
ASV28:
ASV19:
ASV21:

S5a. Pancreatic tumor co-abundance network

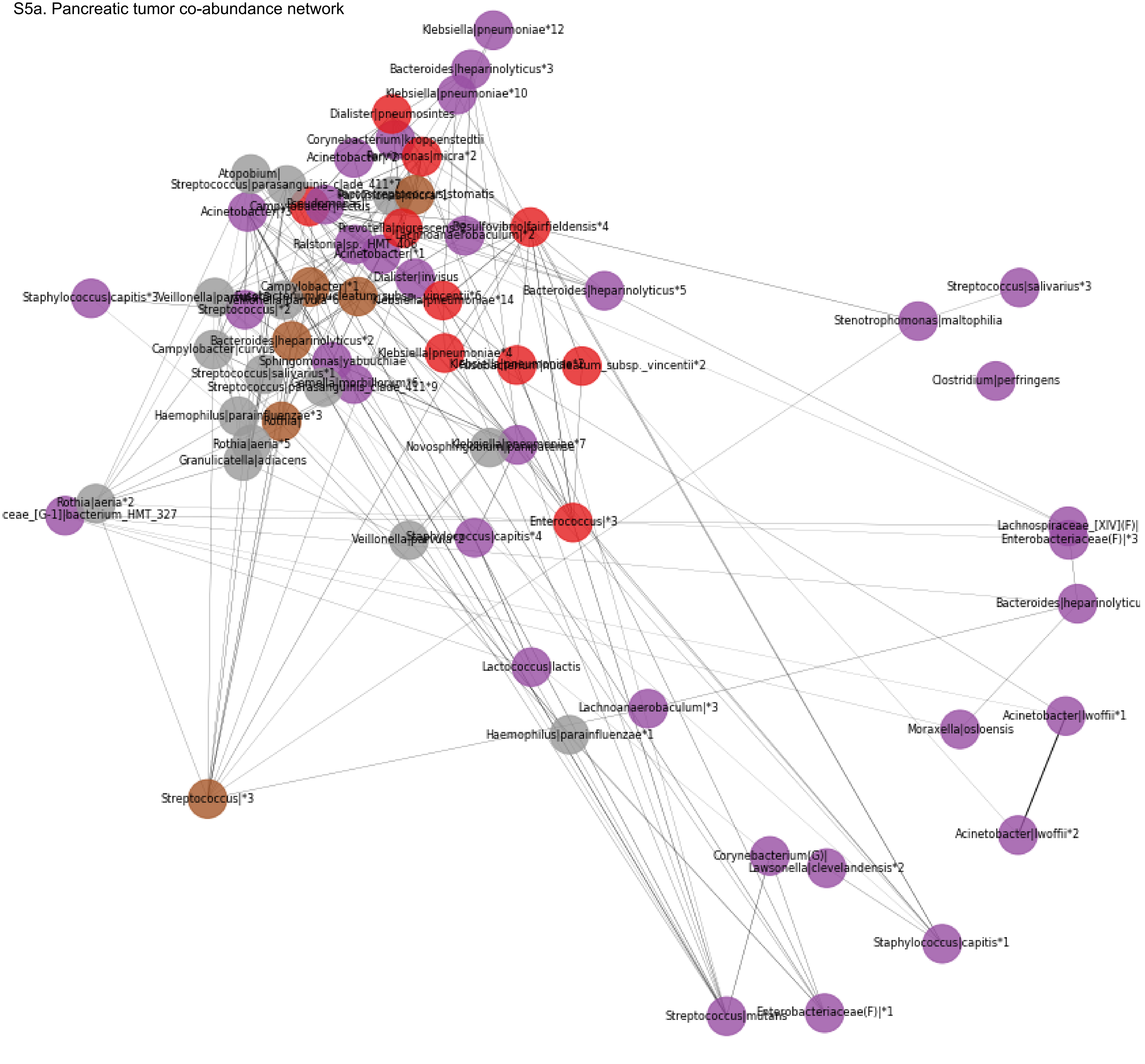

SS5b.

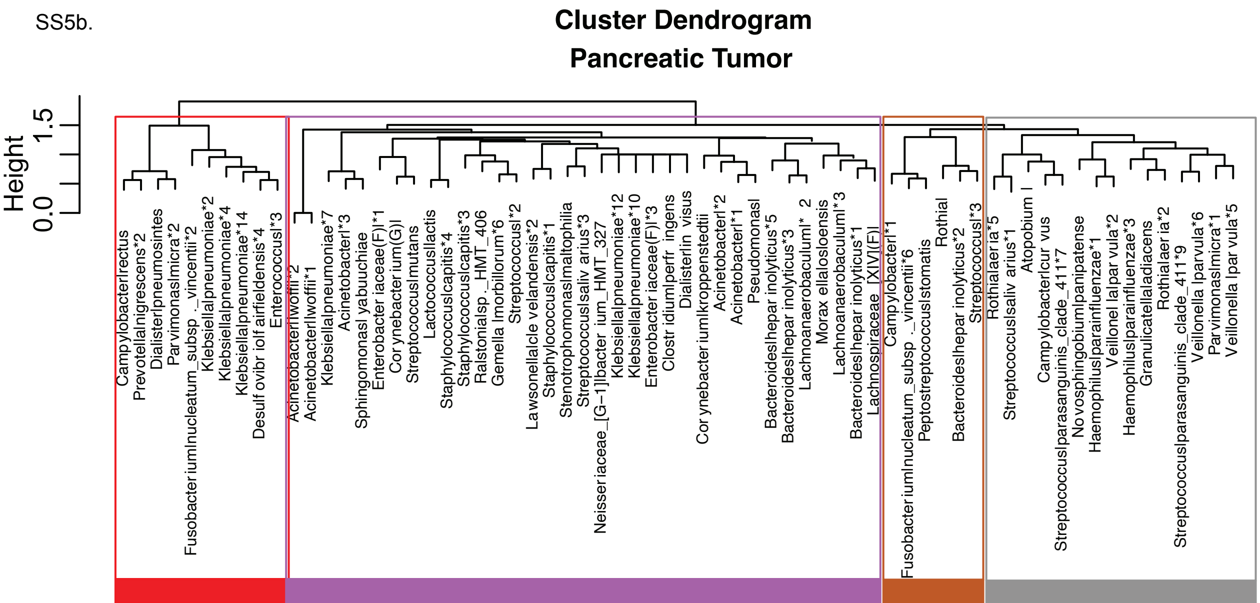
Cluster Dendrogram
Pancreatic Tumor
Height
0.0
1.5
Campylobacter|rectus
Prevotella|nigrescens*2
Dialister|pneumosintes
Parvimonas|micra*2
Fusobacterium|nucleatum_subsp._vincentii*2
Klebsiella|pneumoniae*2
Klebsiella|pneumoniae*4
Klebsiella|pneumoniae*14
Desulfovibrio|fairfieldensis*4
Enterococcus|*3
Acinetobacter|lwoffii*2
Acinetobacter|lwoffii*1
Klebsiella|pneumoniae*7
Acinetobacter|*3
Sphingomonas|yabuuchiae
Enterobacteriaceae(F)|*1
Corynebacterium(G)|
Streptococcus|mutans
Lactococcus|lactis
Staphylococcus|capitis*4
Staphylococcus|capitis*3
Ralstonia|sp._HMT_406
Gemella|morbillorum*6
Streptococcus|*2
Lawsonella|cleavelandensis*2
Staphylococcus|capitis*1
Stenotrophomonas|maltophilia
Streptococcus|salivarius*3
Neisseriaceae_[G-1]|bacterium_HMT_327
Klebsiella|pneumoniae*12
Klebsiella|pneumoniae*10
Enterobacteriaceae(F)|*3
Clostridium|perfringens
Dialister|invisus
Corynebacterium|kroppenstedtii
Acinetobacter|*2
Acinetobacter|*1
Pseudomonas|
Bacteroides|heparinolyticus*5
Bacteroides|heparinolyticus*3
Lachnoanaerobaculum|*2
Moraxella|osloensis
Lachnoanaerobaculum|*3
Bacteroides|heparinolyticus*1
Lachnospiraceae_[XIV](F)|
Campylobacter|*1
Fusobacterium|nucleatum_subsp._vincentii*6
Peptostreptococcus|stomatis
Rothia|
Bacteroides|heparinolyticus*2
Streptococcus|*3
Rothia|aeria*5
Streptococcus|salivarius*1
Atopobium|
Campylobacter|curvus
Streptococcus|parasanguinis_clade_411*7
Novosphingobium|panipatense
Haemophilus|parainfluenzae*1
Veillonella|parvula*2
Haemophilus|parainfluenzae*3
Granulicatella|adiacens
Rothia|aeria*2
Streptococcus|parasanguinis_clade_411*9
Veillonella|parvula*6
Parvimonas|micra*1
Veillonella|parvula*5
