# Supplementary material for: Comparisons of oral, intestinal, and pancreatic bacterial microbiomes in patients with pancreatic cancer and other gastrointestinal diseases
Source: J Oral Microbiol. 2021 Feb 14;13(1):1887680. doi: 10.1080/20002297.2021.1887680 (PMC7889162; doi:10.1080/20002297.2021.1887680)
Supplement: Supplemental Material [file ZJOM_A_1887680_SM1924.zip › Supplementary files/S2 Table.docx]

**S2 Table. Shared ASVs by sites**

| **ASV ID** | **Buccal**  **(n=46)** | **Supra-gingival**  **(n=35)** | **Saliva**  **(n=48)** | **Tongue**  **(n=52)** | **Duo-denum**  **(n=22)** | **Jejunum**  **(n=34)** | **Bile duct**  **(n=19)** | **Panc duct**  **(n=21)** | **Panc nor-mal**  **(n=6)** | **Panc tumor**  **(n=33)** |
| --- | --- | --- | --- | --- | --- | --- | --- | --- | --- | --- |
| ASV01 | 1 | 1 | 1 | 1 | 1 | 1 | 1 | 1 | 1 | 1 |
| ASV02 | 1 | 1 | 1 | 1 | 1 | 1 | 1 | 1 | 1 | 1 |
| ASV03 | 1 | 1 | 1 | 1 | 1 | 1 | 1 | 1 | 1 | 1 |
| ASV04 | 1 | 1 | 1 | 1 | 1 | 1 | 1 | 1 | 1 | 1 |
| ASV05 | 1 | 1 | 1 | 1 | 1 | 1 | 1 | 0 | 1 | 1 |
| ASV06 | 1 | 1 | 1 | 1 | 0 | 1 | 0 | 0 | 0 | 1 |
| ASV07 | 1 | 1 | 1 | 1 | 0 | 1 | 1 | 1 | 0 | 1 |
| ASV08 | 1 | 1 | 1 | 1 | 1 | 1 | 1 | 0 | 1 | 1 |
| ASV09 | 1 | 1 | 1 | 1 | 0 | 1 | 0 | 0 | 0 | 1 |
| ASV10 | 1 | 1 | 1 | 1 | 0 | 1 | 0 | 0 | 1 | 1 |
| ASV11 | 1 | 1 | 1 | 1 | 1 | 1 | 0 | 0 | 0 | 1 |
| ASV12 | 1 | 1 | 1 | 1 | 0 | 1 | 1 | 1 | 0 | 1 |
| ASV13 | 1 | 0 | 1 | 1 | 1 | 1 | 0 | 0 | 1 | 1 |
| ASV14 | 1 | 1 | 1 | 1 | 1 | 1 | 1 | 0 | 0 | 1 |
| ASV15 | 1 | 1 | 1 | 1 | 0 | 1 | 1 | 0 | 0 | 0 |
| ASV16 | 1 | 1 | 1 | 1 | 0 | 1 | 0 | 0 | 1 | 0 |
| ASV17 | 1 | 1 | 1 | 1 | 0 | 1 | 0 | 0 | 0 | 0 |
| ASV18 | 1 | 1 | 1 | 1 | 0 | 1 | 0 | 0 | 1 | 1 |
| ASV19 | 1 | 1 | 1 | 0 | 0 | 1 | 1 | 0 | 0 | 0 |
| ASV20 | 0 | 0 | 1 | 1 | 1 | 1 | 0 | 0 | 0 | 0 |
| ASV21 | 1 | 1 | 1 | 1 | 0 | 1 | 0 | 0 | 0 | 0 |
| ASV22 | 1 | 1 | 0 | 0 | 1 | 1 | 1 | 1 | 1 | 1 |
| ASV23 | 1 | 1 | 1 | 1 | 0 | 1 | 0 | 0 | 0 | 1 |
| ASV24 | 1 | 1 | 1 | 1 | 0 | 1 | 1 | 0 | 0 | 0 |
| ASV25 | 1 | 1 | 1 | 1 | 0 | 1 | 0 | 0 | 0 | 1 |
| ASV26 | 0 | 0 | 1 | 1 | 0 | 1 | 0 | 1 | 0 | 1 |
| ASV27 | 1 | 1 | 1 | 1 | 0 | 1 | 0 | 0 | 0 | 0 |
| ASV28 | 1 | 1 | 1 | 1 | 1 | 0 | 0 | 1 | 0 | 1 |
| ASV29 | 1 | 0 | 1 | 1 | 1 | 1 | 1 | 0 | 1 | 0 |
| ASV30 | 1 | 0 | 1 | 0 | 0 | 1 | 0 | 0 | 0 | 0 |
| ASV31 | 1 | 1 | 1 | 1 | 1 | 1 | 1 | 0 | 0 | 1 |
| ASV32 | 1 | 1 | 1 | 1 | 1 | 1 | 0 | 0 | 0 | 0 |
| ASV33 | 1 | 0 | 1 | 1 | 0 | 1 | 0 | 0 | 1 | 1 |
| ASV34 | 0 | 0 | 0 | 1 | 0 | 1 | 0 | 0 | 0 | 0 |
| ASV35 | 1 | 1 | 1 | 1 | 1 | 1 | 1 | 1 | 0 | 1 |
| ASV36 | 0 | 0 | 1 | 1 | 0 | 1 | 0 | 0 | 0 | 0 |
| ASV37 | 1 | 1 | 1 | 1 | 1 | 1 | 1 | 1 | 0 | 1 |
| ASV38 | 1 | 1 | 1 | 1 | 0 | 1 | 0 | 0 | 0 | 1 |
| ASV39 | 1 | 1 | 1 | 0 | 0 | 1 | 0 | 0 | 0 | 0 |
| ASV40 | 1 | 0 | 1 | 1 | 1 | 1 | 0 | 0 | 0 | 0 |
| ASV41 | 1 | 1 | 1 | 1 | 0 | 1 | 0 | 0 | 0 | 1 |
| ASV42 | 1 | 1 | 1 | 1 | 0 | 1 | 0 | 0 | 0 | 0 |
| ASV43 | 1 | 1 | 1 | 1 | 0 | 1 | 0 | 0 | 0 | 0 |
| ASV44 | 1 | 1 | 1 | 1 | 0 | 1 | 0 | 0 | 0 | 0 |
| ASV45 | 0 | 0 | 0 | 1 | 0 | 1 | 0 | 0 | 0 | 0 |
| ASV46 | 1 | 0 | 1 | 1 | 0 | 1 | 0 | 0 | 0 | 0 |
| ASV47 | 1 | 1 | 1 | 0 | 1 | 1 | 0 | 1 | 1 | 1 |
| ASV48 | 1 | 0 | 0 | 0 | 0 | 1 | 0 | 0 | 0 | 1 |
| ASV49 | 1 | 0 | 0 | 1 | 0 | 1 | 1 | 0 | 0 | 0 |
| ASV50 | 1 | 1 | 1 | 1 | 1 | 1 | 1 | 1 | 0 | 1 |
| ASV51 | 1 | 1 | 1 | 1 | 0 | 1 | 0 | 0 | 0 | 0 |
| ASV52 | 0 | 0 | 1 | 1 | 0 | 1 | 0 | 0 | 0 | 0 |
| ASV53 | 0 | 1 | 0 | 0 | 1 | 0 | 0 | 0 | 0 | 1 |
| ASV54 | 1 | 1 | 1 | 1 | 0 | 1 | 1 | 0 | 1 | 0 |
| ASV55 | 1 | 1 | 0 | 0 | 0 | 0 | 1 | 0 | 0 | 0 |
| ASV56 | 1 | 0 | 1 | 1 | 0 | 1 | 0 | 0 | 0 | 0 |
| ASV57 | 1 | 0 | 1 | 1 | 0 | 1 | 0 | 0 | 0 | 0 |
| ASV58 | 1 | 0 | 1 | 1 | 0 | 1 | 0 | 0 | 0 | 0 |
| ASV59 | 1 | 0 | 1 | 1 | 0 | 1 | 0 | 0 | 0 | 0 |
| ASV60 | 1 | 1 | 0 | 1 | 1 | 0 | 0 | 0 | 0 | 0 |
| ASV61 | 1 | 1 | 1 | 1 | 0 | 1 | 0 | 0 | 0 | 1 |
| ASV62 | 1 | 0 | 1 | 1 | 1 | 1 | 0 | 0 | 0 | 0 |
| ASV63 | 1 | 0 | 1 | 1 | 0 | 1 | 0 | 0 | 0 | 0 |
| ASV64 | 0 | 0 | 1 | 1 | 0 | 1 | 0 | 0 | 0 | 0 |
| ASV65 | 1 | 1 | 1 | 1 | 0 | 1 | 0 | 0 | 0 | 0 |
| ASV66 | 1 | 0 | 1 | 1 | 0 | 1 | 0 | 0 | 0 | 0 |
| ASV67 | 1 | 1 | 1 | 1 | 1 | 0 | 1 | 1 | 0 | 0 |
| ASV68 | 1 | 0 | 1 | 1 | 0 | 1 | 0 | 0 | 0 | 0 |
| ASV69 | 0 | 0 | 1 | 1 | 0 | 1 | 0 | 0 | 0 | 0 |
| ASV70 | 1 | 1 | 0 | 0 | 1 | 1 | 1 | 1 | 0 | 1 |
| ASV71 | 1 | 0 | 0 | 0 | 0 | 1 | 0 | 0 | 0 | 0 |
| ASV72 | 0 | 1 | 1 | 1 | 0 | 1 | 0 | 0 | 0 | 0 |
| ASV73 | 0 | 0 | 0 | 1 | 0 | 1 | 0 | 0 | 0 | 0 |

1 = presence; 0 = absence; panc = pancreatic
