# Supplementary material for: Comparisons of oral, intestinal, and pancreatic bacterial microbiomes in patients with pancreatic cancer and other gastrointestinal diseases
Source: J Oral Microbiol. 2021 Feb 14;13(1):1887680. doi: 10.1080/20002297.2021.1887680 (PMC7889162; doi:10.1080/20002297.2021.1887680)
Supplement: Supplemental Material [file ZJOM_A_1887680_SM1924.zip › Supplementary files/Suppl Material 1 rv2.docx]

**Supplemental Material 1: Sampling, DNA extraction and 16S rRNA gene amplicon sequencing**

**Sampling, DNA extraction and 16S rRNA gene amplicon sequencing**

Data on participants’ demographics and behavioral factors were collected using a self-administered questionnaire, and pancreatic tissue samples and gastrointestinal swabs were collected during surgery using DNA-free forensic sterile swabs whenever possible to reduce contamination . A protocol was established for processing tissue samples collected during surgery to reduce contamination (all tissues were obtained from open surgeries). A technician from the Pathology Department was informed in advance of the surgery date and time, and was paged as soon as the specimens had been obtained. Surgical tissue samples were frozen within 1 hour of the surgery time, as well as tissues swab samples from the stomach, jejunum, and bile duct that were collected using DNA-free forensic sterile swabs whenever possible. During surgery, the surgeon also recorded (on a surgery form for the study) if the patient had received prior pre-OP endoscopic ultrasound (EUS), had previously had their gallbladder removed, or had received prior placement of a stent (for treatment of symptoms); all subjects received a single dose of perioperative antibiotics immediately prior skin incision at the time of the operation. Tissue samples (pancreatic tumors, pancreatic cysts, normal pancreas, pancreatic ducts and duodenums) were prepared by a Rhode Island Hospital pathologist, taking care to avoid contaminating any of the samples; cancerous and non-cancerous tissues were identified and labeled. Unfortunately, we do not have details on percent of tumor content (vs non-tumor) in the specimens prepared by the pathologist and labeled as “tumor” specimens. For those patients who underwent a Whipple procedure (pancreaticoduodenectomy), we considered tissue samples that were located far from the identified tumors and had no apparent tumor presence (e.g., duodenum sections) as “non-tumor”.

Oral swabs were collected from participants prior to surgery using sterile cytology brushes which were immediately placed in tubes containing 700 ul RNA later solution after collection. Saliva was collected using saliva kits (OMNIgene OM-501, DNA Genotek) and processed as per manufacturers’ instructions.

All samples were de-identified and stored at -80ºC until processing for DNA extraction. Nucleic acids were extracted and purified using the DNeasy Blood and Tissue, QIAamp UCP, and QIAamp DNA stool (QIAGEN, Hilden, Germany) for oral cavity and tissues, tissue-swabs, and stool samples respectively, according to the manufacturer’s instructions with minor modifications (addition of an enzymatic lysis step using lysozyme and protein solubilization step using a guanidine thiocyanate buffer). Hypervariable regions of the 16S rRNA gene were sequenced using primers targeting the V3-V4 as paired-end reads on an Illumina platform.

The sequences of each sample were rarefied to 1200 to even the difference in sequencing depth across both oral and intestinal samples for further analysis. The choice of 1200 as sampling depth was guided by reviewing an alpha rarefaction curve that tested various depths ranging between 500 and 5,000. (Figure S1)

**Figure. Alpha Rarefaction Curve**


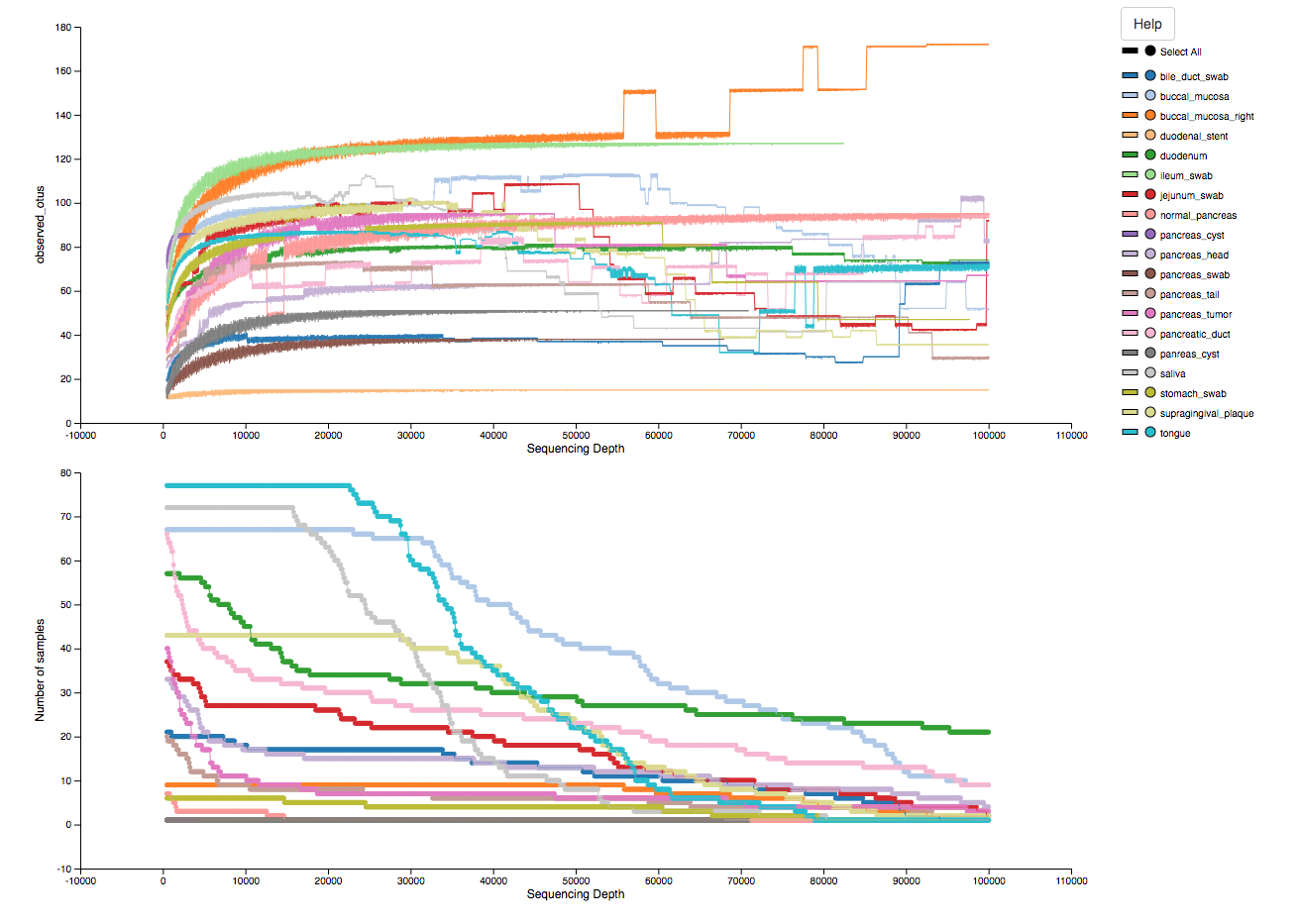


ASV
