# Supplementary material for: Comparisons of oral, intestinal, and pancreatic bacterial microbiomes in patients with pancreatic cancer and other gastrointestinal diseases
Source: J Oral Microbiol. 2021 Feb 14;13(1):1887680. doi: 10.1080/20002297.2021.1887680 (PMC7889162; doi:10.1080/20002297.2021.1887680)
Supplement: Supplemental Material [file ZJOM_A_1887680_SM1924.zip › Supplementary files/Suppl material 2 Statistial Analyses.docx]

**Supplemental material 2: Statistical analyses**

**Pairwise Stratified Association (PASTA) Test**

To conduct the PASTA test, *p*, ω, and μ were first estimated in each site-comparison-by-disease-status subgroup based on a Bayesian Zero-inflated Beta regression model, which was fit utilizing the OpenBUGS statistical analysis software and the R package “R2OpenBUGS” (1). This regression model accounted for within-subject correlations due to repeated measurements via the inclusion of a subject-specific random intercept term. Using the subgroup estimates of *p*, ω, and μ, the posterior probability of exhibiting no association (PN) between the two site-comparison groups was calculated. This probability can be understood as a rejection threshold, where a value of less than or equal to 0.05 was considered statistically significant (i.e., strong evidence of association) and a value of less than 0.1 was considered as marginally significant (i.e., moderate evidence of association).

**ASV co-abundance network**

Force-directed algorithms simulate correlation coefficient values as basic physical properties (2). Positive and negative correlations are modeled as gravity and repulsion, respectively. ASVs with strong positive correlations between each other are plotted near each other, while negatively correlated ASVs are pushed away from each other. In the co-occurrence network plots, each node represented a unique bacterial ASV. Lines between nodes represented correlations between the nodes they connect; with line width indicated the correlation magnitude. Nodes were colored according to their final co-abundance group assignment, calculated using Ward clustering algorithm described earlier. During graphing simulation, the forces were applied to the nodes, pulling them together or pushing them further apart. This iterative process continued until the system reached equilibrium state (lowest total energy) and the relative position of nodes stopped changing from one iteration to the next.

Only ASVs with an absolute SparCC correlation value greater than 0.1 and a p-value less than 0.05, were plotted. Although ASVs belonging to a co-abundance group does not necessarily imply mutual relationships between them, ASVs in the same co-abundance cluster are likely to respond to environment changes in the same fashion (3). In the co-abundance network diagrams, ASVs are colored according to the co-abundance clustering group that they belong to, while their relative positions, ie., being close together or being opposite end of diagram, represent ASVs’ abundance being directly or inversely correlated, respectively.

References:

1. Sturtz S, Ligges U, Gelman AE. R2WinBUGS: a package for running WinBUGS from R. 2005.

2. Fernandez M, Riveros JD, Campos M, Mathee K, Narasimhan G. Microbial" social networks". BMC genomics. 2015;16:S6.

3. Zhang C, Yin A, Li H, Wang R, Wu G, Shen J, et al. Dietary Modulation of Gut Microbiota Contributes to Alleviation of Both Genetic and Simple Obesity in Children. EBioMedicine. 2015;2:968-84.
