# Supplementary material for: Comparisons of oral, intestinal, and pancreatic bacterial microbiomes in patients with pancreatic cancer and other gastrointestinal diseases
Source: J Oral Microbiol. 2021 Feb 14;13(1):1887680. doi: 10.1080/20002297.2021.1887680 (PMC7889162; doi:10.1080/20002297.2021.1887680)
Supplement: Supplemental Material [file ZJOM_A_1887680_SM1924.zip › Supplementary files/Suppl material 3 PASTA rv1.docx]

Supplemental Material 3: PASTA Analyses

The PASTA test identified 2 ASVs (ASV13 and ASV21), *Gemella morbillorum* and genus *Streptococcus*, that showed consistent presence or absence patterns between oral and intestinal or pancreatic samples, after adjusting for within-subject correlation and disease status. For both ASVs, the probabilities of absence tended to be highest among “C24” subjects, second highest among “C25” subjects and lowest among subjects assigned to the “other” or K86.2 group (**Figure S3**). The ASV corresponding to *Gemella morbillorum* also showed a marginally significant association (PN<0.1) for the mean relative abundance (μ). When disease status was coded into three groups (vs 4 groups), similar findings were shown for the two ASVs in the above analysis. Furthermore, three additional ASVs also showed significant (ASV28: *Fusobacterium nucleatum subsp. vincentii*) or marginally significant (ASV67: *Veillonella parvula/dispar*; ASV19: *Streptococcus parasanguinis clade 411*) associations between oral and pancreatic tissue or intestinal samples with respect to *p* (**Figure S3**). None of the ASVs tested showed significant associations with regards to the non-zero mean relative abundance (ω). Detailed PASTA test results of these five ASVs are shown in **Table S3**.

**Figure S3. ASVs that exhibited associations between oral samples and pancreatic tissue or intestinal samples after adjusting for disease types and within-subject correlation structure.** Disease types were grouped into 4 groups in panel a, and were grouped into 3 groups in panel b. Taxonomic annotations of the ASVs are provided on top of each result plot. Legends: panc or intestinal = pancreatic and intestinal samples; PN = posterior probability of there being no association (PN<0.1 is moderate evidence, PN<0.05 is strong evidence); TP evaluates existence of a linear relationship; TS evaluates the existence of a directional relationship (i.e. consistency of rankings). Plots depict parameter estimates (blue diamond or red box) and their 95% credible intervals. For results of the site-specific mean relative abundance (mu), the observed data (blue or red stars) are also plotted next to the intervals.


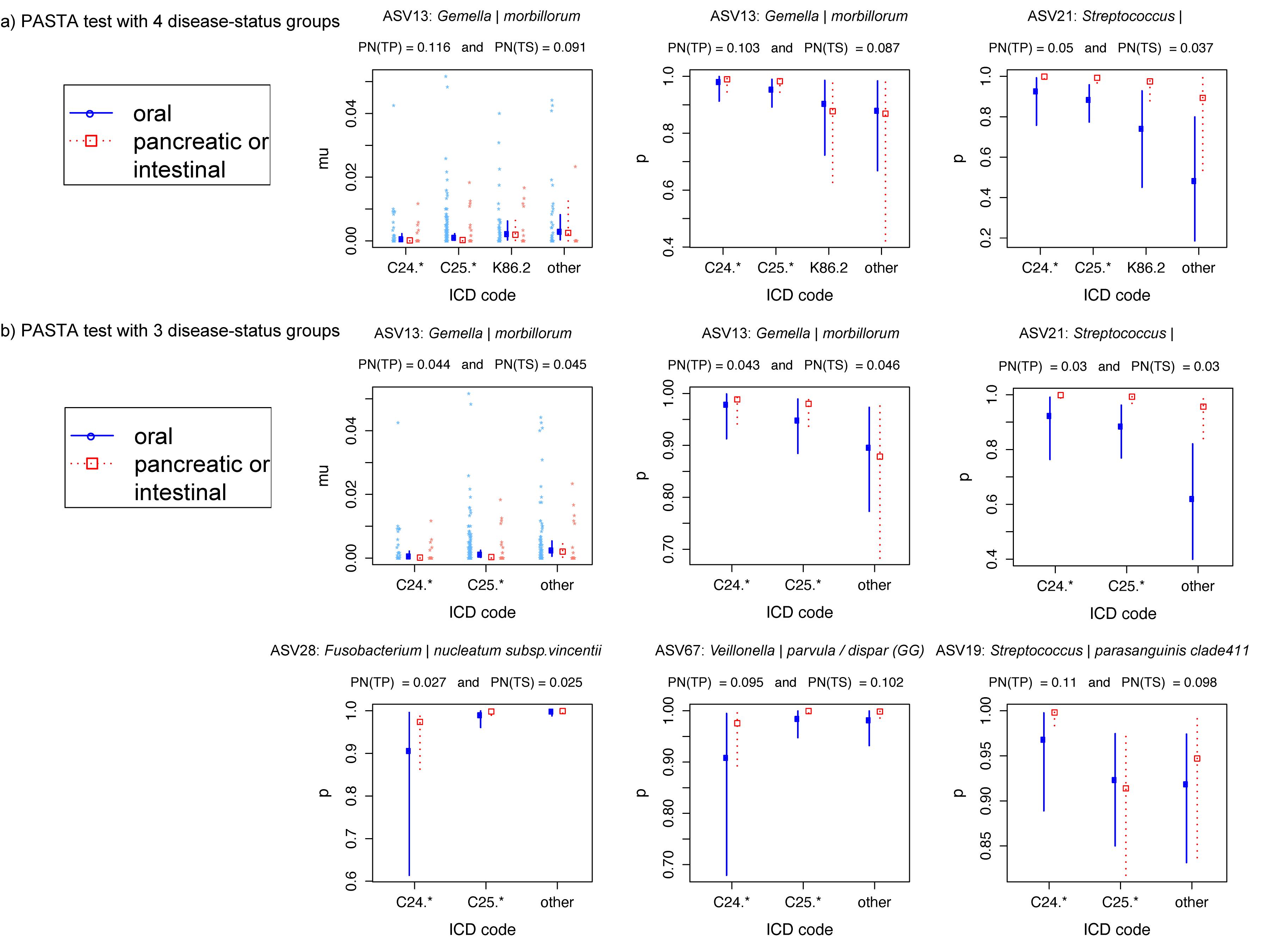


**Table S3. Results of the PASTA test when coding disease status into three groups (“C24.*”, “C25.*”, “other”).** Five ASVs showed consistent patterns between oral samples and pancreatic or intestinal samples with regards to the probability of absence (*p*), non-zero mean relative abundance (*ω*), or mean relative abundance (*μ*). The estimated posterior probability of there being no association (PN) between oral samples and pancreatic or intestinal samples is provided in two forms: TP evaluates existence of a linear relationship and TS evaluates the existence of a directional relationship (i.e. consistency of rankings). PN < 0.1 represents moderate evidence of a consistent pattern and PN < 0.05 represents strong evidence of a consistent pattern.

| **ASV ID^b^** | **PN**  **μ**  **TP** | **PN**  **μ**  **TS** | **PN**  **ω**  **TP** | **PN**  **ω**  **TS** | **PN**  **p**  **TP** | **PN**  **p**  **TS** | **Genus^a^** | **Species (HOMD)^a^** |
| --- | --- | --- | --- | --- | --- | --- | --- | --- |
| ASV28 | NA | NA | NA | NA | 0.0274 | 0.025 | *Fusobacterium* | *nucleatum subsp. vincentii* |
| ASV21 | NA | NA | NA | NA | 0.03 | 0.03 | *Streptococcus* |  |
| ASV13 | 0.0444 | 0.045 | 0.4774 | 0.4744 | 0.0432 | 0.0462 | *Gemella* | *morbillorum* |
| ASV19 | NA | NA | NA | NA | 0.11 | 0.0976 | *Streptococcus* | *parasanguinis clade 411* |
| ASV67 | NA | NA | NA | NA | 0.0952 | 0.102 | *Veillonella* | *parvula* |

NA = not applicable; PN posterior probability of there being no association (PN<0.1 is moderate evidence, PN<0.05 is strong evidence); TP evaluates existence of a linear relationship; TS evaluates the existence of a directional relationship (i.e. consistency of rankings)

^a^ Taxonomic annotations of the ASVs are from HOMD database unless otherwise noted.

^b^ ASV ID used in the Figure 1 left panel.
